# Data-driven identification of outpatient-suitable procedures: a machine learning approach

**DOI:** 10.1007/s10729-026-09758-6

**Published:** 2026-03-23

**Authors:** Robert Messerle, Jonas Schreyögg

**Affiliations:** 1 Hamburg Center for Health Economics, Universität Hamburg, Esplanade 36, 20354 Hamburg, Germany; 2 Wissenschaftliches Institut der AOK (WIdO), Berlin, Germany

**Keywords:** Policy, Healthcare services, Ambulatory surgical procedures, Hospital economics, Capacity planning

## Abstract

Policymakers worldwide are encouraging a shift from inpatient to outpatient care to improve the efficiency of health systems. One of the first steps in such efforts typically involves allowing providers, often hospitals, to perform a designated list of procedures on an outpatient basis. However, determining which procedures are suitable for the hospital outpatient setting remains challenging and has traditionally relied on expert judgment and established practices. Our study advances this approach by employing supervised machine learning techniques to identify patterns in physician and expert decisions. We present a comprehensive classification of hospital procedures as either inpatient- or outpatient-suitable and use some methods of explainable AI methods to identify the main factors influencing these assessments. Our model achieves high accuracy (92%) and a robust area under the receiver operating characteristic curve (> 95%), assigning outpatient suitability scores to the entire German procedure catalog. These scores can easily be used with aggregate hospital data from different countries. To validate our approach, we applied the model to a surgical procedures shortlist from the OECD. Our findings provide decision-makers with a data-driven framework for developing targeted strategies to incentivize the provision of outpatient care.

**Supplementary Information:**

The online version contains supplementary material available at 10.1007/s10729-026-09758-6.

## Highlights

- We developed a machine learning classification model to enable the data-driven identification of medical procedures that may be suitable for outpatient settings.
- Our model assigns outpatient suitability scores, using data from 115 million hospital cases.
- Using some methods of Explainable AI, the model shows the extent to which different variables influence the outpatient suitability scores of individual procedures, supporting informed decision-making by stakeholders.
- Our study suggests that when evaluating the suitability of a medical procedure for outpatient provision, decision makers should prioritize the time from procedure to discharge for specific patient groups rather than focusing solely on the overall length of stay for all patients.
- The model equips decision makers with tools to develop targeted strategies that incentivize hospitals to optimize their service delivery.

**Supplementary Information:**

The online version contains supplementary material available at 10.1007/s10729-026-09758-6.

## Introduction

Driven by advances in medical technology and a desire to improve healthcare efficiency recent years have seen a strong international trend towards shifting procedures that were once exclusively performed in hospitals as inpatients to outpatient settings. Health policymakers worldwide are promoting this transition, whether by investing in outpatient hospital infrastructure or encouraging a greater role for office-based providers, depending on country-specific organization of specialist care [1–3].

Nevertheless, OECD data [4] indicate substantial variation across countries: while Scandinavia and English-speaking countries, routinely provide many surgical procedures on a hospital outpatient basis, countries in Central and Eastern Europe, such as Germany or Poland, continue to rely on inpatient care. Even countries that have made substantial progress in this area, such as Denmark, continue to implement policies aiming to reduce inpatient care volume [5].

These ongoing reforms have important implications for various operational aspects of hospitals, such as resource allocation, surgical scheduling, and appointment management [6–8]. One challenge at the heart of these reforms is identifying which cases are suitable for outpatient care. A question that must be answered on two levels. Firstly, at the macro level a definition of the procedures generally considered for outpatient care must be established. Based on this, the medical context of individual cases can be discussed in depth at the micro level, taking into account factors such as the risks faced by individual patients, and incorporating this information into a clinical decision support system. While the scientific literature on identifying suitable patients for outpatient care is extensive [9–11], to the best of our knowledge, no studies have examined how to identify suitable procedures or conditions for outpatient settings at a macro level. This is a knowledge gap that our study aims to address.

In many countries, inpatient and outpatient care are defined by regulatory lists, either constraining outpatient procedures to specific codes (as in Germany) or identifying procedures that can only be billed as inpatient admissions (as in the US Medicare system). Several countries, including Italy, France, and the United Kingdom, promote hospital outpatient care through target shares, recommended rates, or financial incentives for selected procedures [12–14].

Existing approaches to determining suitable outpatient procedures often rely on expert panels and standards of practice. However, these methods can be slow, lack transparency, and may not systematically address key predictors of outpatient suitability [12, 15]. To address these limitations, a data-driven approach is needed to replace or at least supplement the heuristic, experience-based, and subjective decision-making process with methods and reasoning that are understandable, justifiable, and actionable. Our approach employs machine learning techniques trained on expert judgements and incorporating predictors reflective of current hospital treatment processes. Unlike traditional full black box models, our approach leverages some aspects of explainable AI and attributable analytics, quantifying the effect of individual factors at the procedure level. Although we do not develop a fully explainable AI model [16], our approach enhances interpretability and supports broader stakeholder acceptance and policy application.

Our approach allows for the specification of outpatient suitability levels for all procedures, providing decision-makers with a comprehensive basis for developing outpatient/inpatient procedure lists. By using the predictions of our model, they are not solely dependent on expert consensus. The approach is easily adaptable to different data, allowing for consideration of national contexts, such as differences in hospital processes. A data-driven assessment can also guide further stakeholder discussions. Identifying the importance of the individual factors driving these designations, such as patient morbidity or length of stay, can serve as the foundation for evidence-based decision-making.

Our proposed classification model contributes to several areas of the scientific literature. First, to the best of our knowledge, it represents the first algorithmic approach to determining the outpatient potential of medical procedures at the macro level. Our model therefore facilitates the identification of procedures that could particularly benefit from financial incentives [12].

Second, our study adds to the growing body of literature on the application of machine learning, particularly using some aspects of explainable AI, in decision-making and health economics [16–24]. Our approach enhances the transparency compared with existing approaches and can complement existing decision frameworks for outpatient suitability classification.

Third, and perhaps most importantly, our results can substantially improve capacity planning for outpatient and inpatient resources by enabling more accurate estimation of the potential of procedures to be performed in an outpatient setting. Predicting demand for healthcare resources is a recurring theme in health economics and operations research [25–31]. An important aspect of this process is understanding the characteristics of the patient population, particularly the distinction between elective and non-elective (emergency) cases, as well as between inpatient and outpatient care [32, 33]. Many studies have examined specific aspects of capacity planning, such as inpatient care [29, 34] or elective procedures [35, 36]. Due to the differing nature of the planning processes involved they often do not to consider that these processes are interdependent. For example, the unpredictable arrival of emergency patients often disrupts the scheduling of inpatient services. Outpatient services, despite disruption from higher no-show rates, therefore tend to offer more efficient resource utilization, such as operating room use, potentially freeing up capacity for additional care [37]. Accurate capacity planning thus requires reliable estimates of these probabilities. For emergency cases, a data-driven, diagnosis-based classification has been suggested, enabling the integration of urgency probabilities into emergency care planning [38]. Our study contributes to capacity planning by presenting a data-driven, algorithmic approach to assess the outpatient suitability of various procedures, thereby generating information that can be systematically included in planning processes.

Finally, our model can improve estimates of responses to price changes by specifying cross-elasticities of care. Several studies have identified considerable cross-elasticities between outpatient and inpatient services [39, 40]. By predicting the potential for outpatient provision, our classification allows for a more systematic estimation of the responses to price changes in both sectors.

## Hospital outpatient care in Germany

Historically, hospital services in Germany have been almost entirely restricted by law to inpatient settings. Even today hospitals remain sharply focused on inpatient care, with revenues from inpatients accounting for over 90% of total revenues [41], although they are now permitted to perform certain procedures on an outpatient basis. The ‘AOP catalogue’ outlines these procedures. This catalogue is negotiated between health insurers and representatives of hospitals and office-based physicians, who provide the majority of specialist outpatient care in Germany. The negotiations determine the scope of procedures suitable for outpatient treatment in hospitals. The catalogue underwent only minimal changes in the years before 2020, mainly involving administrative coding updates. It was only from 2020 onwards that new procedures were incorporated, all of which had previously been designated as inpatient procedures. These new codes may therefore not reflect the same level of consensus regarding their outpatient suitability as earlier codes. An overview of the procedure groups and the corresponding number of codes covered by the AOP-catalogue across different updates can be found in Appendix Table (6). Until 2023, the catalogue categorised procedures into two groups: category 1, comprising services that should generally be provided on an outpatient basis at a hospital, and category 2, comprising services suitable for either outpatient or inpatient care.

However, various factors, such as a lack of post-acute care options or communication challenges, could be used by hospitals to justify inpatient treatment. Given these factors, along with the strong financial incentives associated with higher inpatient payments, it is no surprise that hospitals in Germany continue to treat the majority of patients who are undergoing procedures included in the outpatient catalog as inpatients (approximately two thirds in 2019 [42]). Due to this historically limited adoption of hospital outpatient care, the German hospital system offers a unique context for developing a robust outpatient classification model. Although we use a German adaptation of the International Classification of Procedures in Medicine (ICPM) to develop our model, we aggregate the resulting outpatient score at the ICD level, allowing for compatibility with a widely used international classification system. We also provide a slimmed-down model available online for further analysis.

### Data

We aggregated administrative data from the German diagnosis-related group (DRG) system, covering all inpatient episodes in Germany from 2014 to 2019 at the procedure code level. The DRG dataset included detailed case-level information, including admission and discharge times by hospital department, primary and secondary diagnoses, and time-stamped OPS procedure codes (a German modification of the International Classification of Procedures in Medicine, ICPM). Additionally, it included patient demographics such as age and sex, covering 115 million inpatient episodes. Due to the case-based nature of the dataset, patient linkages over time were not possible. To enable the identification of hospital readmissions, as well as the use of outpatient emergency services and prescriptions, we supplemented the aggregated DRG data with aggregate claims data from 43 statutory health insurers in Germany. This additional data covered the years 2014 to 2019 and approximately eight million insured individuals, representing about 10% of the population. Procedures performed fewer than 100 times annually in Germany were excluded from our analysis.

### Feature engineering/predictor variables

Feature engineering is essential for ensuring that the model has sufficient information to achieve high predictive power. This process involves extracting input, or predictor variables from the data that are relevant to the outcome of interest and the model being employed. To maintain consistency with our outpatient classification, we used data from 2014 to 2019 to compute relevant features for the training and test datasets, thereby minimizing the potential impact of the COVID-19 pandemic on our analysis. From a dataset of approximately 115 million hospital cases during this period, we generated 178 features, of which 118 were used in the final model.

The features of the algorithm were derived from a number of perspectives, as shown in Table 1. Tables 4 and 5 in the Appendix provide a comprehensive list of all feature definitions and descriptive statistics.

**Table 1:** Overview of features used in classification

Perspective of features	Description
Complications	This perspective captures short-term adverse outcomes following hospital discharge such as readmissions
Comorbidities	This perspective quantifies the burden of coexisting conditions using the Elixhauser comorbidity index, age and individual diagnoses
Assistance	This perspective reflects the level of post-discharge support and long-term care needs such as the share of patients discharged to nursing homes
Additional Procedures	This perspective captures the volume, timing, and type of procedures performed during hospitalization in addition to the index procedure such as the share of patients receiving imaging at night
Urgency	This perspective reflects the urgency of hospital admissions, mainly based on a validated classification model from Krämer et al. (2019)
Case Timestamps	This perspective describes the temporal patterns of hospital admissions and discharges such as the average length of stay
Procedure Timestamps	This perspective focuses on the timing of procedures in relation to admission and discharge such as the average time from procedure to discharge
Departments	This perspective captures the complexity and dynamics of intra-hospital care pathways such as the average number of departments involved in the case
Diagnoses	This perspective reflects the diagnostic complexity associated with procedures
Ventilation	This perspective quantifies the use and variability of mechanical ventilation

A major source for identifying appropriate features was previous work involving interviews with healthcare experts. In addition, we used literature on patient selection for day surgery [2] as well as studies in the health care domain dealing with classification problems, e.g. on length of stay prediction [38, 43, 44].

In Germany, current discussions about expanding day surgery currently tend to focus on the length of stay as an indicator of outpatient suitability. Important perspectives were therefore process and case timestamps as well as values derived from these. These included the length of stay as well as many other time-related features such as the share of procedures performed at night time or at weekends. As with all other perspectives, all characteristics were aggregated information, such as means, variances, percentiles or proportions relative to the total population of cases. More than one aggregation was usually included (such as average and percentiles or average and variance) to provide as much information as possible for the classification. Normally, a procedure is performed as part of a larger hospital stay involving multiple procedures. We therefore also included features covering information on additional procedures such as imaging or medication and their timings. In line with the literature on patient selection for day surgery, we included proxies for patients' comorbidities, which are important when deciding on patient pathways [45]. We used Elixhauser comorbidities, age and additional information, such as functional impairment or the need for long-term care. In order to determine whether a procedure is performed for specific diagnoses or is used for a wide range of conditions, we calculated the number of different primary and secondary diagnoses and corresponding concentration measures (Herfindahl–Hirschman index). To identify the medical complexity of a procedure, we included several characteristics related to the resources involved. We calculated the number of days spent in pre- and post-hospital care, as well as the proportion of transfers to other departments and the timing of these transfers. Finally, we used information on patients' need for mechanical ventilation.

Our approach provided a broad range of features to assess outpatient suitability that are relevant from both the demand and supply perspectives. All features were continuous and standardized as z-scores for use in the model. Any missing observations were imputed by mean. Although tree-based methods are generally robust to highly correlated features [46, 47]. Nonetheless, we applied a correlation filter during feature preprocessing as an additional precaution.

### Learning and test dataset

To train our algorithm and evaluate its performance, we needed a dataset with an existing classification of outpatient and inpatient procedures.

For our analysis, we included all category 1 procedures from pre-2020 versions of the AOP-catalog as the basis for outpatient training and test assignments. Both classifications, inpatient and outpatient, should be interpreted with caution. Because procedures from both lists can still be provided in either inpatient or outpatient settings depending on the circumstances, these classifications should be seen as indicating the preferred setting for these procedures. They do not represent a strict dichotomy like the clear distinction between alive and dead used in survival analysis, but rather a guideline for determining the most appropriate setting for each procedure.

Out of 31,109 procedures for which feature data were available, 1,247 procedures were classified as outpatient and 5,060 classified as inpatient.^1^ After applying a filter for frequent procedures, defined as those with 100 or more cases per year, 1,130 outpatient procedures and 640 non-outpatient/inpatient procedures remained from a reduced total of 11,285 procedures in our training set. Thus, the training data was slightly imbalanced with a ratio of 2:1, which we deemed a mild imbalance without taking further steps [48, 49].

As test for generalization, we applied our classification model to updates of the AOP catalog after 2020 and used a recent, extensive proposal of potentially outpatient-suitable procedures from a policy report [42]. Under the assumption that these procedures have a high potential for outpatient provision, our model should predict high outpatient suitability scores.

## Methods

**Fig. 1:**
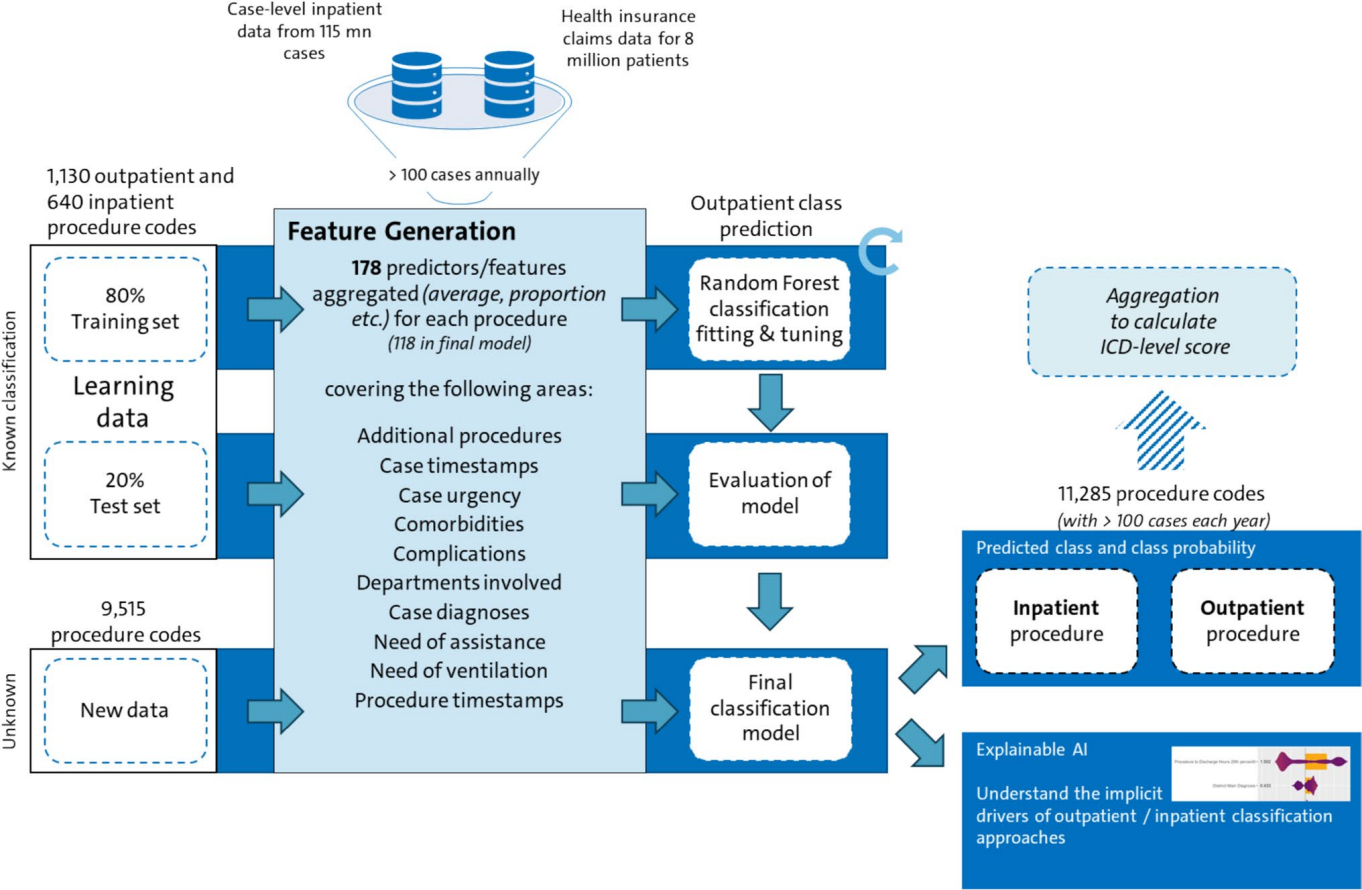
Overview of methodological approach

An overview of our methodological approach is shown in Fig. 1.

### Classification algorithm

Classification problems are a major branch of supervised learning, and a wide range of machine learning techniques have been developed for this purpose in recent years. A very common and effective approach is classification trees and their ensembles/extensions [50], such as random forests [51] or boosted trees/gradient boosting machines [52].

We used random forest [51, 53], a bagging algorithm that frequently outperforms other machine learning techniques [54].

The performance of the model was evaluated using the area under the receiver operating characteristic (ROC) curve (AUC), which is closely related to the Gini coefficient. The ROC curve illustrates the true-positive rate versus the false-positive rate across a range of classification thresholds and is used to depict the tradeoff between these indicators. The AUC is a widely used summary metric of the ROC curve and provides a threshold-independent performance measure that accounts for all possible sensitivity–specificity tradeoffs. [55]. We used an 80/20 training-test split for training and the final model evaluation using random sampling. The final ROC AUC value was thus calculated on data never used in training. To tune the external parameters (i.e., hyperparameters) of our model we used grid search with a size of 100 and tenfold cross validation within the training data set. Table 8 in the Appendix contains more information.

We also evaluated other common machine learning classification techniques: LASSO [56], XGBoost [57], (multilayer perceptron) neural networks [58], support vector machines [59, 60], and naïve Bayes classification [61]. Random forests and XGBoost demonstrated comparable performance, and both outperformed the other techniques on our dataset based on cross-validation. We selected random forests for our final model because it is considered to be slightly less prone to overfitting [62].

Our approach results in each procedure code being assigned a class probability. Because Germany uses a localized version of the International Classification of Procedures in Medicine (ICPM), the results are not directly applicable to other contexts. However, we provide a slimmed-down version of our machine learning model online [link: https://osf.io/nqfse/overview?view_only=0c6389feddac438ea4a8868dfec716bd], which can be applied to other datasets. To further facilitate the use of our results, we also transformed the procedure-based score into a diagnosis-based score. This involved assigning two potential scores to each inpatient case: the lowest procedure-based outpatient suitability score and the average of all procedure-based scores. We then calculated the diagnosis-based score by averaging these two outpatient suitability scores for all cases associated with a given primary diagnosis.

### Model explainability and interpretation

In machine learning, high predictive power is often associated with low interpretability [63]. Several methods have been proposed to overcome this trade-off and help interpret and explain complex models and their predictions ([16, 64] provide an overview). Broadly speaking, these methods are categorized into local methods, which explain individual predictions, and global methods, which help explain overall model results.

SHAP values (SHapley Additive exPlanations) [63] are among the most widely used local methods for interpreting the results of machine learning models. They are based on Shapley values [65] from game theory. The Shapley value for a feature j is calculated as follows:

$${\upphi }_{j}\left({f}_{x}\right)={\sum }_{S\subseteq \{1,\dots ,p\}\backslash \{j\}}\frac{\left|S\right|!\left(p-\left|S\right|-1\right)!}{p!}\left[{f}_{x}\left(S\cup \{j\}\right)-{f}_{x}\left(S\right)\right]$$

where p is the number of features and S are all possible subsets of the features not containing feature j [64]. For each subset, the difference between the prediction ${f}_{x}\left(S\cup \{j\}\right)$, which includes features S and j, and ${f}_{x}\left(S\right)$, which includes only features S, is computed. The Shapley value is the average difference over all possible combinations, representing the marginal contribution of feature j to the individual predictions. The sum of all Shapley values equals the total effect of the features on the prediction made by the model. Due to their high computational cost, exact Shapley values are not feasible for most machine learning applications. From among the several algorithms available to efficiently approximate Shapley values [64, 66], we used fastshap [67, 68]. However, because SHAP values are additive in the log-odds space, interpreting them at the level of individual predictions is not straightforward. To improve local explanations, we also used the breakdown approach, which is easy to interpret and provides results directly in terms of probabilities [69]. This approach can be understood as an approximation of Shapley values.

The additive nature of SHAP values allows global explanations to be aggregated directly from individual observations. Aggregated SHAP values can be used to rank the importance of each predictor variable for classification, providing a global and abstract view of the model. In addition, SHAP-based dependence plots can be used to show the direction and strength of the influence that each feature has on the predictions made by the model. We also considered other approaches, such as partial dependence plots. The main interpretation remained unchanged, so we focused on SHAP. However, it should be kept in mind when interpreting that different methods can produce different explanations, especially at a local level [70, 71].

To further assess the reliability of SHAP explanations, we evaluated faithfulness metrics based on the OpenXAI framework [72, 73]. Specifically, we computed the Predictive Gap on Important (PGI) and Predictive Gap on Unimportant (PGU) scores by systematically perturbing the more and less influential features, respectively, and measuring the corresponding change in model predictions. Larger PGI relative to PGU indicates that the features identified as important truly affect model behavior.

## Results

### Classification

**Fig. 2:**
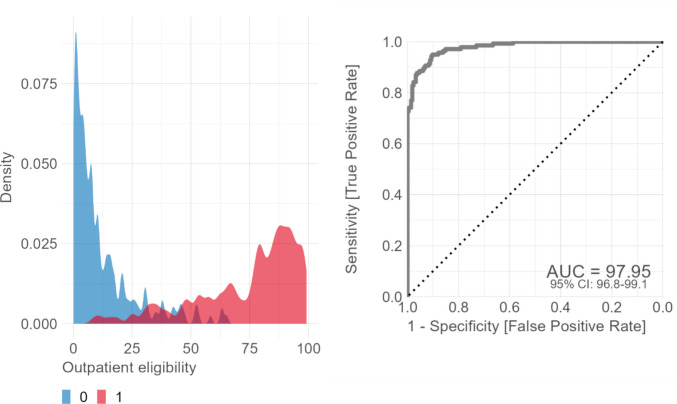
Classification model performance

Among the models tested, XGBoost and random forests performed best, outperforming the alternatives (Appendix Fig. 8). Final tuning of the classification model increased the AUC by only about 0.5% compared to the default parameters (see Appendix Table 8 for exact parameters of the final model). The final classification model performed very well, achieving an AUC of 98% and an accuracy of 92% on the test set, with a sensitivity of 97% and a specificity of 84%. Thus, the final classifier is highly effective at distinguishing between outpatient and inpatient procedures. Figure 2 shows the outpatient suitability estimates and the AUC-ROC plot for the test dataset. Outpatient procedures (outpatient suitability > 50%) had an average score of 80%, whereas inpatient procedures (outpatient suitability ≤ 50%) had an average score of 13%. Twenty-two percent of the test data procedures had a score between 25 and 75%.

Applied to the full dataset of procedures with more than 100 annual cases, our classification model yielded class assignments and class probabilities for 11,285 procedures, covering more than 99% of all relevant inpatient cases (i.e., cases with a procedure code) in Germany. Overall, the average outpatient classification score across these procedures was 39%. Compared to the results from training and testing, we observed a relatively wide distribution of outpatient eligibility scores, spanning the entire range from clear-cut inpatient to clear-cut outpatient procedures (see Appendix Fig. 9).

Table 2 lists the top 10 outpatient procedures, and Table 3 the top 10 inpatient procedures from the entire German procedure catalog. Both tables are based on a pre-filtered list of the top 100 procedures by frequency (roughly 50% of the whole market). Results for all procedures can be found in Online Resource 2.

**Table 2:** Top 10 outpatient procedures (from the top 100 by frequency)

Procedure code	Title	Outpatient prediction	In training data
5–812.5	Arthroscopic surgery on the joint cartilage and menisci: Partial meniscus resection	0.98	
5–144.5a	Extracapsular extraction of the lens [ECCE]: Lens nucleus liquefaction [phacoemulsification] via corneal access: With insertion of a capsule-fixed posterior chamber lens, monofocal intraocular lens	0.96	x
1–653	Diagnostic proctoscopy	0.95	
1–661	Diagnostic urethrocystoscopy	0.95	x
5–932.00	Type of material used for tissue replacement and reinforcement: Non-absorbable material: Without coating	0.94	
3-13d.5	Urography: Retrograde	0.94	
1–444.6	Endoscopic biopsy of the lower digestive tract: Step biopsy	0.93	
1–650.2	Diagnostic colonoscopy: Total, with ileoscopy	0.93	x
1–444.7	Endoscopic biopsy of the lower digestive tract: 1 to 5 biopsies	0.91	x
1–650.1	Diagnostic colonoscopy: Total, up to the cecum	0.91	x

**Table 3:** Top 10 inpatient procedures (from the top 100 by frequency)

Procedure code	Title	Outpatient prediction	In training data
5–820.00	Implantation of a hip joint endoprosthesis: Total endoprosthesis: Uncemented	0.07	
8–981.1	Neurological complex treatment of acute stroke: More than 72 h	0.07	x
5–469.20	Other bowel surgeries: Adhesiolysis: Open surgical	0.09	
5–822.g1	Implantation of a knee joint endoprosthesis: Bicondylar surface replacement prosthesis: Cemented	0.10	
8–931.0	Monitoring of respiration, heart, and circulation with measurement of central venous pressure: Without continuous spectrometric measurement of central venous oxygen saturation	0.14	
8–981.0	Neurological complex treatment of acute stroke: At least 24 to a maximum of 72 h	0.14	x
8–831.0	Insertion and change of a catheter in central venous vessels: Insertion	0.15	
8-98f.0	Complex intensive medical treatment (basic procedure): 1 to 184 resource points	0.18	x
5–986.x	Minimally invasive technique: Other	0.20	
8–980.0	Intensive medical treatment (basic procedure): 1 to 184 resource points	0.20	x

Diagnostic proctoscopy, diagnostic colonoscopy, cataract surgery, and endoscopic biopsy of the lower digestive tract were assigned as clear-cut outpatient procedures. Five out of the top ten procedures were already included in the AOP catalog category 1 and were thus part of our training data. Others, such as a partial meniscus resection, were included as category 2 or added later. Among the top inpatient procedures were the implantation of an endoprosthesis in the hip and knee joints, as well as open surgery adhesiolysis. Additionally, procedures codes representing supplementary information, such as insertion and replacement of a catheter in central venous vessels, were included in the top 10 list.

### Generalizability

Our classification model was trained exclusively on medical procedures that were already considered suitable for outpatient care according to the German AOP-catalogue prior to 2020. In 2023 and 2024, the AOP-catalogue was expanded, incorporating over 200 new codes. In line with the existing AOP-catalogue the update was focussed on surgical procedures spread across a wide range of surgical fields (see Appendix Table 6).

This expansion provided a valuable opportunity to validate the surprisingly good performance of our model in a real-world generalizability test on so far unseen data. Additionally, a comprehensive expert report identified a substantial list of procedures with potential for outpatient care [42]. It included a broader spectrum of possible procedures and took a different approach then the AOP-catalogue. It included also those procedures which could only in some specific contexts be provided as outpatient and did not focus specifically on surgical procedures (see Appendix Table 7).

Both the catalogue extension and the expert report serve as valuable external datasets for evaluating our model. However, neither source provides explicit reasoning for why specific procedures were deemed suitable for outpatient care. This is where our model adds value: it can help uncover the underlying patterns and decision logic that may implicitly inform these classifications.

Based on our model, both datasets were predominantly assigned an outpatient suitability score greater than 0.5 and were thus classified as outpatient procedures (see Appendix Figs. 10 and 11). Importantly, in line with expectations the scores for the AOP-catalogue updates were consistently above 0.5, whereas the scores of the expert lists were more scattered.

### Application to OECD surgical shortlist

**Fig. 3:**
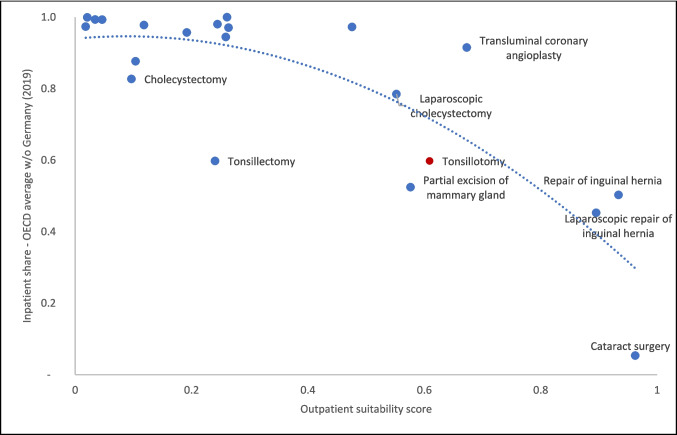
Outpatient suitability scores for OECD surgical shortlist. Notes: Dotted polynomial (order 2) trendline. Red dot: Tonsillotomy is not listed as a separate procedure on the OECD surgical shortlist but is included within the OECD definition of tonsillectomy. Inpatient share for all procedures in Germany was > 90% in 2019 except for cataract surgery (17%)

The OECD surgical shortlist is regularly used for international comparisons of day surgery rates [4]. Using our model, we compared outpatient suitability scores with international progress in outpatient/day surgery provision. Figure 3 plots the average inpatient share across OECD countries (excluding Germany) against the outpatient suitability score for various procedures derived from German inpatient data. Overall, we see a strong correlation between the two variables, indicating that our model based on German inpatient data provided a reasonable representation of the general outpatient potential across OECD countries. This result adds additional credibility to the generalization of our model. Transluminal coronary angioplasty, with a relatively high outpatient score but a high inpatient share, and tonsillectomy, with a low outpatient score but a high outpatient share, appear to be outliers which warranted further analysis.

Transluminal coronary angioplasty was not included in the training data and is mostly performed on an inpatient basis in OECD countries. However, some of the corresponding procedure codes, which account for a large proportion of actual care, have recently been added to the AOP catalog and were already permitted for use by office-based physicians. In addition, the international literature, particularly from the US, suggests that outpatient care may indeed be a safe option for transluminal coronary angioplasty [74–76].

Tonsillectomy is performed on an outpatient basis in approximately 40% of cases across OECD countries, yet our model classifies it as an inpatient procedure. This might result from the OECD definition of tonsillectomy, which encompasses several procedure codes of varying complexity. For Fig. 3 we aggregated all corresponding procedure codes from the OECD definition. More complex procedure codes such as a total tonsillectomy were classified as inpatient by our model, but some of the less extensive procedures in this definition were classified as outpatient, such as a partial tonsillectomy or tonsillotomy, which has an assigned score of 0.60. This is consistent with recent updates to the AOP catalog, in which tonsillotomy was included.

Individual explanations for both procedures are presented in the next section.

### Model interpretation

#### Global interpretation

**Fig. 4:**
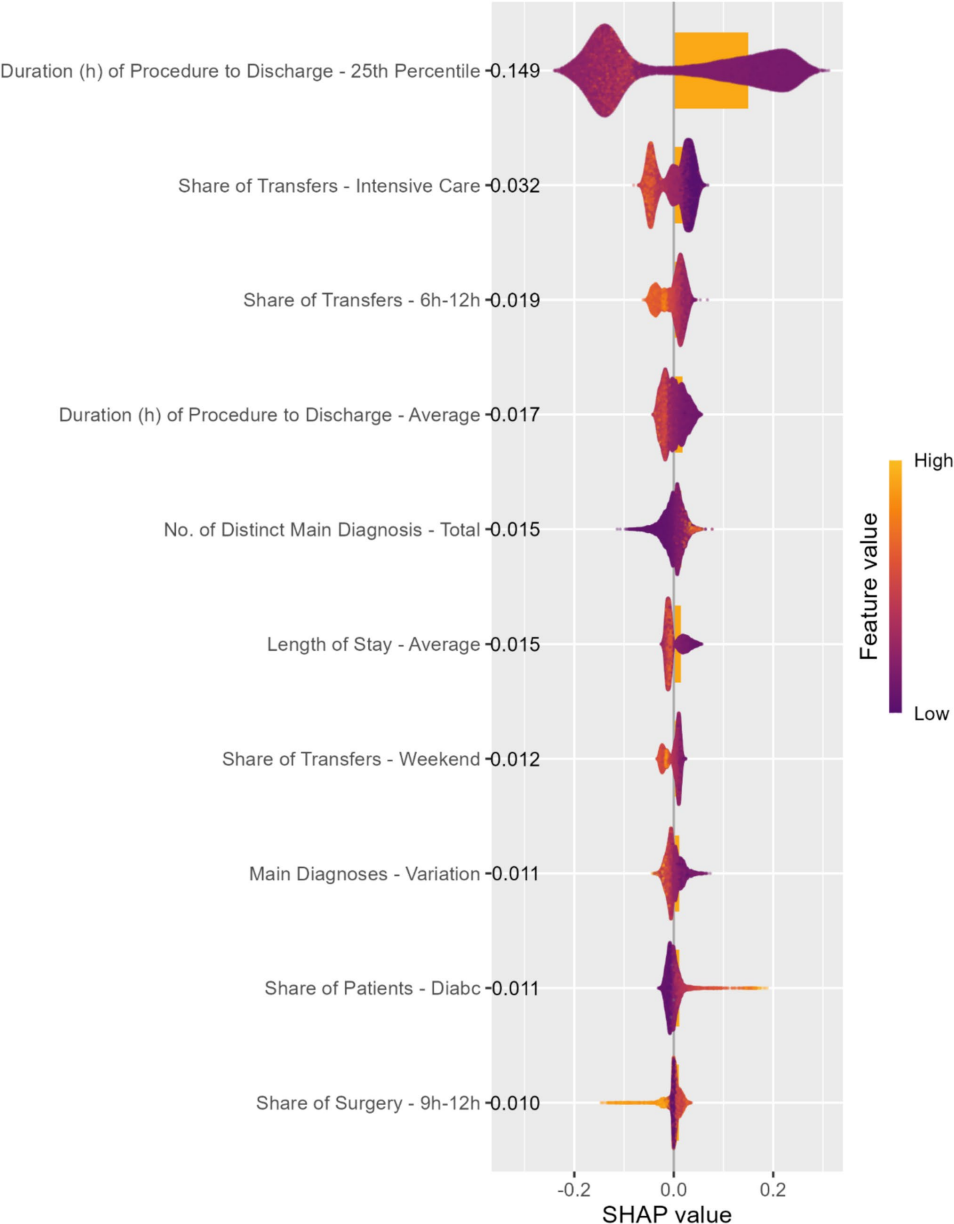
Top 10 feature contributions and SHAP values

Figure 4 plots the individual SHAP values for each feature and observation for the 10 most important features, ranked by their average absolute SHAP values (plotted as bars). The plot also depicts the direction of feature impact. Positive SHAP values increase and negative SHAP values decrease the *log odds* of a positive (i.e., outpatient) classification. The color of the dots indicates whether high or low feature values (e.g., long or short durations) contribute to the corresponding SHAP value.

Figure 4 shows that timing-related features strongly influenced the classification, particularly the 25th percentile of the time from the provision of a procedure to patient discharge. As expected, longer time spans (indicated in orange in Fig. 4) correspond to lower outpatient eligibility. Other important features include transfers between departments and to the ICU, the number of distinct main diagnoses, and the variation in main diagnoses (measured as a Herfindahl–Hirschman index (HHI)/concentration measure) for each procedure code. Only a few comorbidities (based on the Elixhauser comorbidity index), especially complicated diabetes, contribute substantially to the classification.

**Fig. 5:**
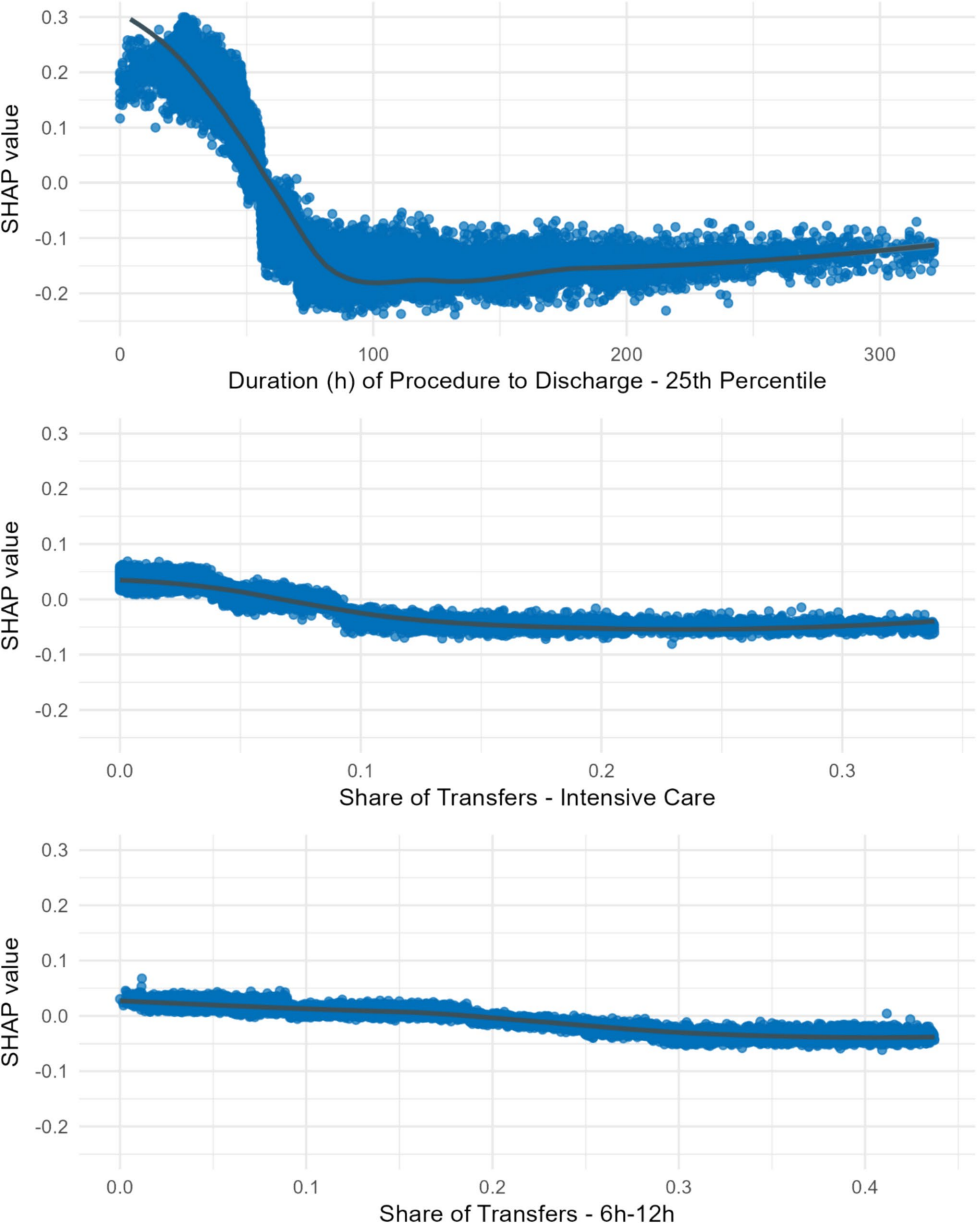
SHAP dependence plot for top three features, only 95-%-quantile plotted

To better understand the dynamics of our classification model Fig. 5 shows how the model relied on the top three features by plotting the SHAP values for each individual observation (i.e., one procedure code) and feature value. The vertical scatter of data points represents interaction effects with other features, resulting in varying impacts for different observations. Figure 5 illustrates that larger 25th percentiles for procedure-to-discharge times initially result in declining outpatient suitability scores. However, the effect stabilizes around 100 h, beyond which further increases do not reduce the scores. Similar patterns can be observed for the share of transfers to intensive care and the share of transfers occurring between 6 a.m. and noon (also shown in Fig. 5). Additional dependence plots for all top 10 features are provided in Online Resource 3.

To evaluate the robustness of the SHAP explanations, we also computed Predictive Gap on Important (PGI) and Predictive Gap on Unimportant (PGU) metrics. PGI values exceeded PGU by a wide margin (mean PGI = 0.28, PGU = 0.04), confirming that the SHAP attributions are faithful to the underlying model’s decision structure.

#### Local interpretation

In addition to illustrating the global importance of variables, recent advancements have enabled local explanations for individual observations, greatly improving the transparency of machine learning results. In this context, we applied our model to two procedure groups from the OECD surgical shortlist.

**Fig. 6:**
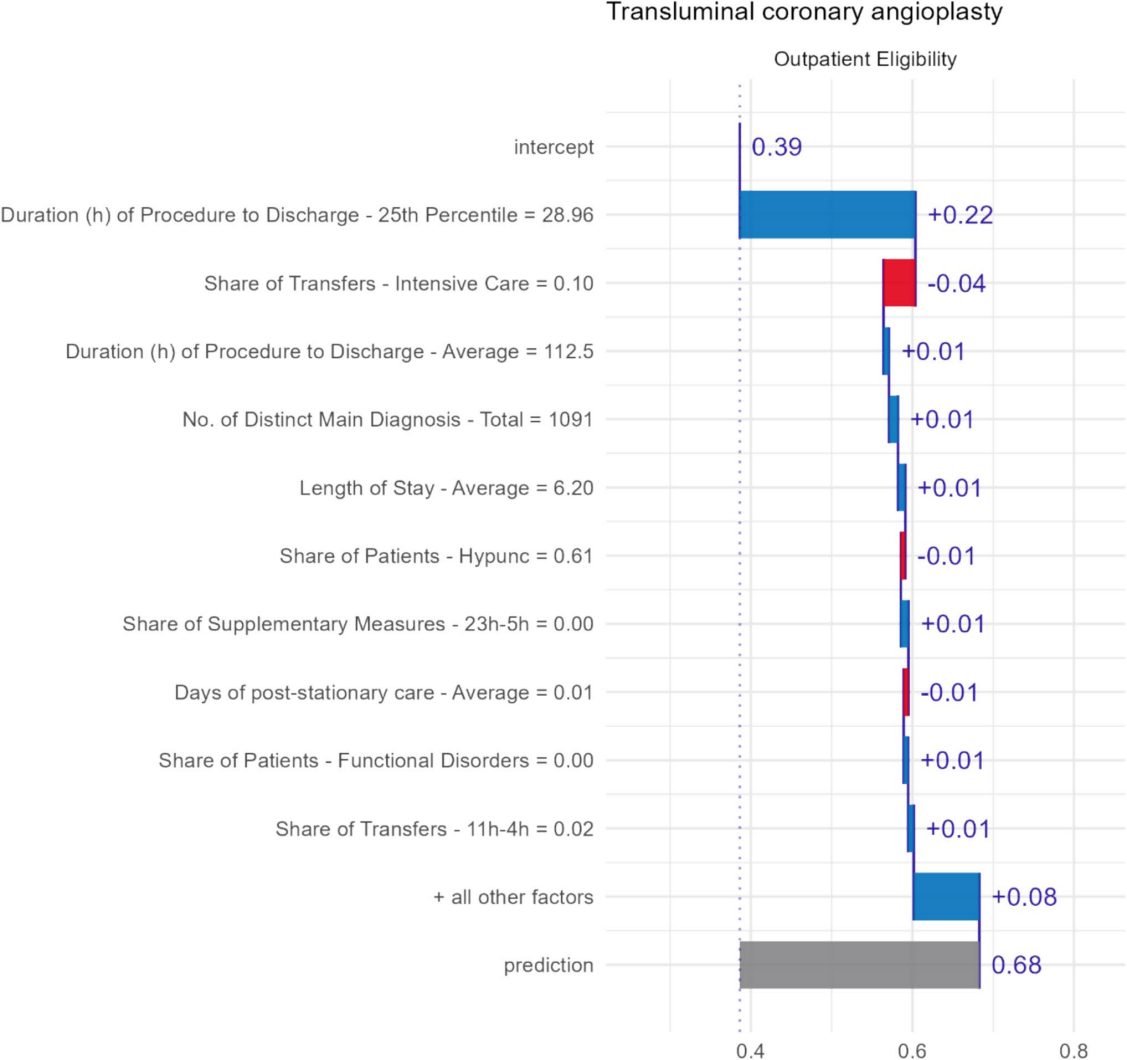
Interpretation of classification based on SHAP values for transluminal coronary angioplasty

Figure 6 provides a breakdown of individual feature contributions for transluminal coronary angioplasty or percutaneous coronary intervention (PCI). According to our model, PCI receives an outpatient suitability score of 68%, classifying it as an outpatient procedure. The feature contributions are markedly concentrated, with the most influential feature being the 25th percentile of the duration from procedure provision to patient discharge, recorded at 29 h. This factor increased the score by 22 percentage points compared to the average of 39%. For reference, in the training data, the average 25th percentile was 162 h for inpatient procedures and 44 h for outpatient procedures. The second most important variable is the share of transfers to intensive care, which, at 10%, decreased the score by 4 percentage points. In comparison, the average rates of transfer to intensive care in the training data were 13% for inpatient procedures and 3% for outpatient procedures. The contributions of all other features are relatively minor, each being less than or equal to 1%, with a cumulative contribution of 12 percentage points.

**Fig. 7:**
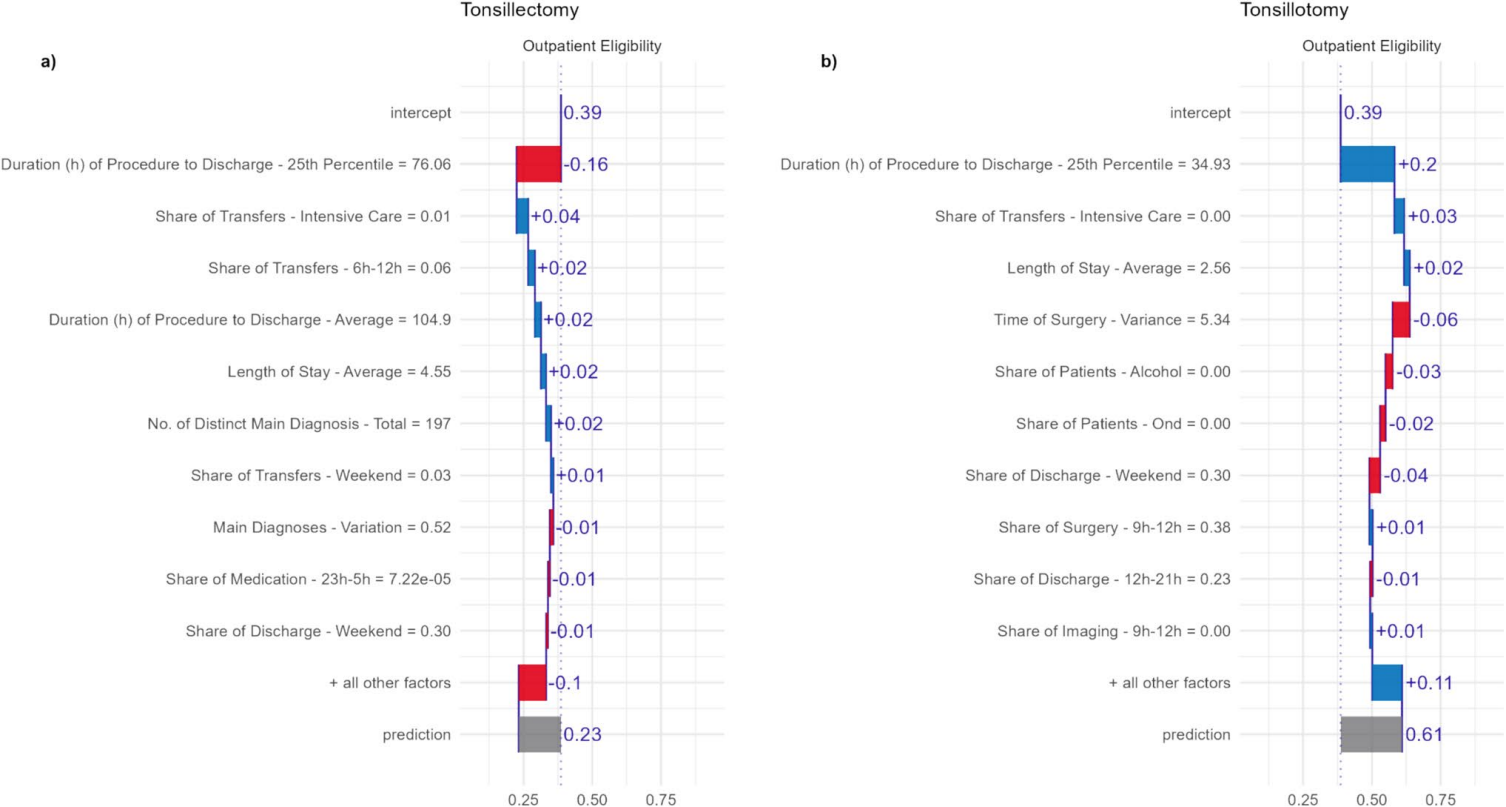
Interpretation of classification based on SHAP values for tonsillectomy and tonsillotomy

Figure 7, part a, presents the feature contributions for tonsillectomy, another procedure group from the OECD surgical shortlist. For comparison, Fig. 7, part b, presents the corresponding contributions for tonsillotomy, a related procedure within the tonsillectomy group. This comparison demonstrates how the model identified data-driven distinctions between closely related procedures. Tonsillectomy receives an outpatient eligibility score of 23%, classifying it as an inpatient procedure. The feature contributions are more dispersed than those for PCI, with the 25th percentile of the procedure-to-discharge time (76 h for tonsillectomy) being the most important factor, reducing the score by 15 percentage points compared to the average. In contrast, tonsillotomy is classified as an outpatient procedure with a score of 61%, which is largely due to a smaller 25th percentile of procedure-to-discharge time of 35 h. Other features play a lesser role in the classification. For tonsillectomy, the share of transfers to intensive care and transfers occurring between 6 a.m. and noon are also important variables. For tonsillotomy, the variance in surgery timing and the share of weekend discharges are relevant features. This comparison highlights how similar feature values can yield different contributions due to feature interactions and non-linear effects. For example, a 1% share of transfers to intensive care increases the outpatient suitability score for tonsillectomy by 4 percentage points, while a 0% value increases the score for tonsillotomy by only 3 percentage points.

## Discussion

Germany provides a unique opportunity for developing an outpatient classification model based on machine learning. In many countries, outpatient-suitable procedures are no longer performed in traditional inpatient settings. As a result, selection between outpatient and inpatient treatment has already occurred, making data preparation much more difficult or even impossible. In contrast, the uptake of outpatient-suitable procedures in the German hospital system has been slow, and data on inpatient and outpatient procedures is comprehensive and well-suited for classification efforts.

Our proposed model demonstrates the effectiveness of our classification approach with its high AUC of > 95% and accuracy of 92%. However, the data description in Appendix Table 4 already shows the substantial differences between the two classes in the training data pointing at the possibility of a clear distinction. This is not surprising, given that the two procedure groups are at opposite ends of the care process spectrum.

Furthermore, it is important to note that our classification problem does not occur on the patient level, so it supposedly does not include a lot of random noise. Instead, it incorporates institutional and expert judgements, which are frequently influenced by average criteria such as length of stay and usual risk of complications. Thus, while an AUC score of 0.9 would be an outstanding result for any medical application, our model essentially 'reverse-engineers' the aggregate decision-making patterns of experts. Using features, each representing an average across thousands of cases per procedure, our model formalises the implicit rules that have guided catalogue development to date.

To assess whether the high ROC AUC reflected genuine signal rather than overfitting to noise, we conducted a permutation test by randomly shuffling the outcome labels in the training data and re-training the model. As expected, the resulting AUC dropped to approximately 0.50, indicating that the original performance was not due to overfitting to noise. Additionally, adding synthetic noise by appending random data columns did not increase model performance or appear in feature importance rankings. Moreover, a simpler model using a logistic regression also achieved a high AUC of 0.9 (see Appendix Fig. 8).

The feature importance analysis suggests that the classification model is plausible, with the leading predictors based on a variety of timestamps and main diagnoses. Because the existing catalog of hospital outpatient procedures is the result of negotiations influenced by expert judgments, our results offer insights into the underlying reasoning. In particular, the duration from the time a procedure is performed to patient discharge appears to be a particularly important factor. Indeed, our findings indicate that the 25th percentile of this variable was the most important feature in determining outpatient suitability. This suggests that a procedure may be considered suitable for hospital outpatient care if timely discharge is possible for a subset of patients, even if it is not feasible for all. Given that the outpatient catalog in Germany defines the list of procedures permitted – not mandated – for hospital outpatient treatment, this finding is in line with our expectations. Other important features, such as the share of transfers to intensive care units, also appear to be consistent with outpatient provision.

The strong predictive power of timing variables suggests that these variables are likely to be central to the original assignment of outpatient suitability. To address the risk of circularity, we conducted an analysis that excluded all case-timing-related features. The model’s performance dropped only slightly and the AUC score remained above 0.9. Instead of variables directly related to timing, other variables, which in turn influence key timing features, such as the share of transfers at the weekend or the number of departments involved, were among the most important features.

Based on our generalizability test, which involved predicting scores for recently proposed additional outpatient procedures, our classification of outpatient and inpatient care appears medically reasonable. Our study thus introduces the first algorithmic approach to systematically determine the outpatient potential of medical procedures, filling a gap in existing research by identifying procedures that should be prioritized for outpatient care. By providing a data-driven model that delivers understandable, justifiable, and actionable results, our approach ensures that stakeholders do not need to blindly trust the results of a black-box model but rather can engage in informed discussions based on comprehensible information.

Our study also contributes to the field of health economics and operations research by providing useful information for capacity planning and predicting demand across healthcare settings. While previous research has often addressed isolated aspects of capacity planning – focusing either on inpatient care or outpatient procedures [29, 34] – our results represent a step towards a data-driven approach. Systematically incorporating a measure of outpatient suitability into capacity planning can support the development of more accurate forecasting models, ultimately improving the utilization of healthcare resources. Although much recent research on capacity planning has concentrated on operational aspects, such as forecasting surgery duration [23], waiting times [24], or bed assignments [27], healthcare decision makers are increasingly focusing on reorganizing service provision [77]. Such initiatives require capacity and demand forecasting models that can provide actionable and explainable insights. Key input factors for such models [25, 30, 78], such as the number of outpatients or the average length of stay, can benefit from a systematically derived outpatient suitability model.

Our results offer several potential applications beyond those discussed above. Because our approach is based on current hospital care practices, it can easily be applied to hospital data in other contexts, provided that key information, such as length of stay and proportion of transfers, is available. Unlike methods that rely on international benchmarks to assess outpatient suitability, our data-driven approach considers the specific context of care provision. It accounts for variations in hospital workflows, which may result in different suitability outcomes for the same procedure across different settings. For example, joint endoprosthesis is mentioned in the literature as a potential outpatient procedure [79]. However, our model classifies it clearly as an inpatient procedure, with a score of 0.1 (see Table 3). Considering that the average length of stay for these cases is roughly 10 days, this assessment seems plausible for the German context. 

Because we provide a slimmed-down version of our model online [link: https://osf.io/nqfse/overview?view_only=0c6389feddac438ea4a8868dfec716bd], no training data or computationally intensive learning or tuning are required. As an additional simplified option, we provide a diagnosis-based (ICD-10) score with averaged outpatient suitability scores for further use in Online Resource 2.

Furthermore, by using lists of procedures, such as the OECD surgical shortlist, our model provides an easy-to-interpret assessment of care processes across different contexts. It can also be used to evaluate explainable and unexplainable regional differences in hospital utilization for outpatient care. Studies have shown substantial variation in hospital outpatient services, which can only be partially attributed to observable patient and hospital characteristics [80, 81]. By incorporating the underlying parameters of hospital care, our approach allows internal processes to be included in these assessments, offering a more comprehensive analysis.

Given the financial strain on healthcare systems in many countries, our findings are particularly relevant for policies aiming to improve the efficiency of healthcare provision. Existing hospital utilization data can be used to assess the efficiency of current care processes – their “outpatient suitability” – and compare them across different levels, such as individual hospitals or regions. Additionally, many OECD countries face non-financial resource constraints, including staffing shortages and limited bed availability. In these contexts, a data-driven, transparent approach to identifying promising areas for incentivizing outpatient care and reducing admissions could be useful for optimizing resource utilization.

Lastly, several studies have identified cross-elasticities between outpatient and inpatient care [39, 40], as well as marked substitution patterns between hospital and GP care [82]. These analyses might be improved by including the “outpatient progress” of individual services. Our results enable more precise estimations of responses to price changes and cross-elasticities with outpatient care, taking into account different levels of outpatient suitability.

Our study has several limitations that need to be considered when interpreting its results. First, our classification does not replace individual medical assessments. It is based on averages derived from existing administrative data and is not appropriate for case-specific prognosis. The methodology is empirical and focuses on classification accuracy rather than causality, making it suitable only as a policy tool for the preliminary identification of potential outpatient procedures and target values. Moreover, our approach used data from 2014 to 2019. While this was appropriate for training the data due to its harmonisation with the AOP catalogue, predictions from our model should be updated using more recent data to provide an up-to-date prediction of outpatient potential.

Second, although our analysis of SHAP scores provides important explanations for our results, these should be used only as a basis for further discussion, not for drawing final conclusions. While our approach enhances model interpretability, we acknowledge that this represents only a limited aspect of Explainable AI [16]. Robust explainability extends beyond post-hoc feature analysis to include other aspects such as an explicit decision-making logic [83] and uncertainty quantification in the model’s predictions [84, 85]. Moreover, future research should take into account different methodological approaches, such as using proxy methods to account for collinearity [86] or making use of new insights regarding Shapley values [87]. Lastly, since our label data is influenced by administrative and policy rules our model formalizes current conventions and cannot guarantee to highlight new or causally superior predictors.

In summary, this study illustrates the broad potential of supervised machine learning as a tool for policymakers and healthcare managers, who often require such formalisations. Although a wide range of applications in healthcare have been presented in recent years, most have involved case-specific predictions, specific diseases, or individual medical decision making [18, 88]. We show that machine learning is also a valuable policy tool at a more abstract, macro level.

## Conclusion

This study presents, to the best of our knowledge, the first comprehensive framework for classifying and categorizing the outpatient suitability of medical services. Our model assigns varying levels of outpatient suitability to each procedure in the German procedure catalog and is easily adaptable to hospital data in different countries. By improving the understanding of how outpatient procedures are identified, this framework enables policymakers, healthcare managers, and hospital administrators to develop targeted strategies that encourage hospitals to optimize their provision of health care services. Such strategies can help reduce unnecessary admissions and allow hospitals to better allocate resources for essential inpatient treatments.

## Electronic supplementary material

Below is the link to the electronic supplementary material.

Supplementary file1 (CSV 15 KB)

Supplementary file2 (XLSX 323 KB)

Supplementary file3 (PDF 2287 KB)

## Figures and Tables

**Fig. 8:**
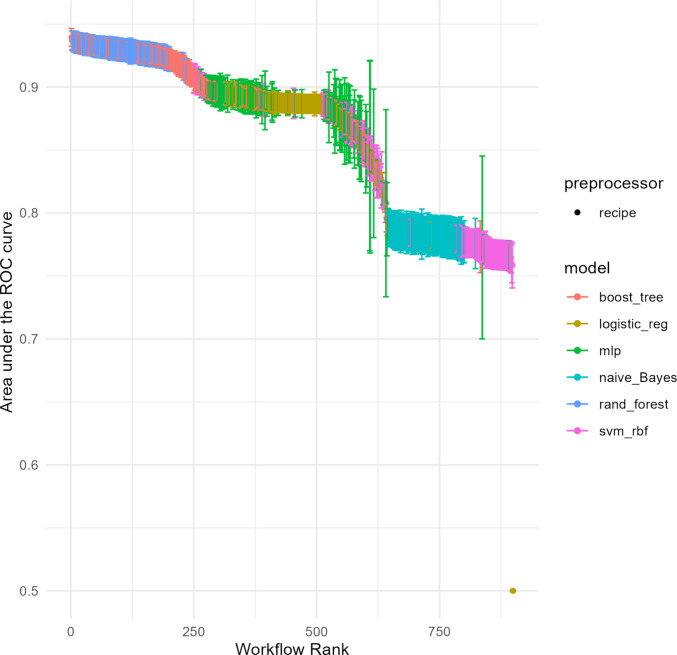
Model comparison for 150 model parametrizations based on tenfold cross validation for each

**Fig. 9:**
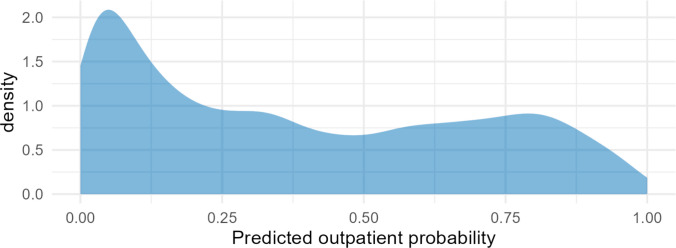
Distribution of outpatient suitability scores for all procedures of German OPS catalog

**Fig. 10:**
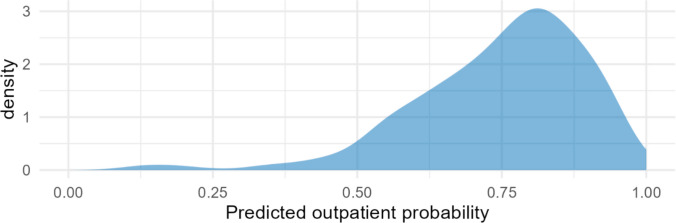
Distribution of outpatient suitability scores for all new procedures codes of the AOP catalog added after 2023 catalog

**Fig. 11:**
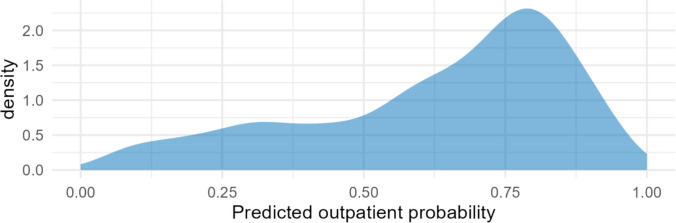
Distribution of outpatient suitability scores for all proposed procedure codes from consultants (Gutachten §115b SGB V)

**Fig. 12:**
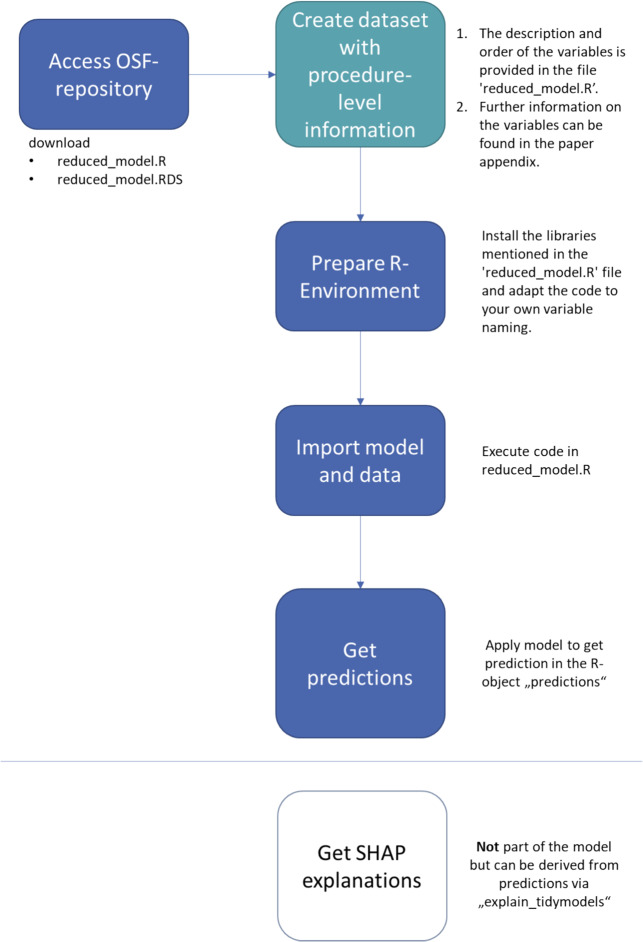
Flowchart for use of reduced model

## Data Availability

Data used for training the model is not available due to data protection restrictions.

## Appendix

### Description of data preprocessing and tuning

Preprocessing and tuning of the data was done with tidymodels (https://www.tidymodels.org/) in R 4.4. Ranger was used as package for the random forest. The original split was 80:20 for training and test data set. The following preprocessing steps were used:

1. Removing all predictors with zero variance (0 were removed)
2. Mean-imputing all numeric predictors
3. Normalizing all numeric predictors
4. Filtering variables with a high correlation (> 0.9).

Further tuning was performed on the training dataset using tenfold cross-validation with random sampling and no stratification using grid search with a size of 100 candidate parameter combinations (using Space-filling parameter grids). All model metrics were then calculated on the test set, which had not been seen until then.

**Table 4:** Summary of feature variables

Summary of feature variables (unweighted mean of procedure code averages)		
	Inpatient	Outpatient
Complications		
Share of Patients—Painkiller (30-day)	0.16	0.16
Share of Patients—Outpatient Emergency (30-day)	0.11	0.08
Share of Patients—Readmission (30-day)	0.14	0.09
Share of Patients—In-hospital Mortality	0.04	0.01
Comorbidities		
Elixhauser Score—Average	5.01	2.15
Share of Patients—CHF	0.09	0.04
Share of Patients—Carit	0.14	0.09
Share of Patients—Valv	0.05	0.02
Share of Patients—PCD	0.02	0.01
Share of Patients—PVD	0.06	0.04
Share of Patients—Hypunc	0.37	0.30
Share of Patients—Hypc	0.03	0.02
Share of Patients—Para	0.03	0.02
Share of Patients—Ond	0.05	0.03
Share of Patients—CPD	0.07	0.05
Share of Patients—Diabunc	0.11	0.08
Share of Patients—Diabc	0.03	0.03
Share of Patients—Hypothy	0.11	0.08
Share of Patients—RF	0.10	0.06
Share of Patients—LD	0.03	0.01
Share of Patients—PUD	0.00	0.00
Share of Patients—AIDS	0.00	0.00
Share of Patients—Lymph	0.00	0.01
Share of Patients—Metacanc	0.04	0.02
Share of Patients—Solidtum	0.13	0.06
Share of Patients—Rheumd	0.02	0.02
Share of Patients—Coag	0.10	0.03
Share of Patients—Obes	0.09	0.07
Share of Patients—Wloss	0.04	0.01
Share of Patients—Fed	0.20	0.06
Share of Patients—Blane	0.00	0.00
Share of Patients—Dane	0.02	0.01
Share of Patients—Alcohol	0.02	0.01
Share of Patients—Drug	0.01	0.00
Share of Patients—Psycho	0.01	0.00
Share of Patients—Depre	0.05	0.03
Age—Average	56.33	53.15
Share of Patients—Functional Disorders	0.03	0.01
Assistance		
Share of Discharges to Hospice, Rehab, Nursing	0.08	0.02
Share of Patients—Nursing Assistance Level 1	0.01	0.01
Share of Patients—Nursing Assistance Level 2	0.04	0.02
Share of Patients—Nursing Assistance Level 3	0.03	0.02
Share of Patients—Nursing Assistance Level 4	0.01	0.01
Share of Patients—Nursing Assistance Level 5	0.00	0.00
Additional Procedures		
Number of Procedures—Average	9.22	5.69
Share of Diagnostics—23 h-5 h	0.02	0.01
Share of Imaging—23 h-5 h	0.03	0.01
Share of Surgery—23 h-5 h	0.05	0.04
Share of Medication—23 h-5 h	0.00	0.00
Share of Non-surgical Therapeutics—23 h-5 h	0.10	0.03
Share of Supplementary Measures—23 h-5 h	0.02	0.01
Share of Diagnostics—9 h-12 h	0.13	0.07
Share of Imaging—9 h-12 h	0.20	0.09
Share of Surgery—9 h-12 h	0.49	0.42
Share of Medication—9 h-12 h	0.01	0.00
Share of Non-surgical Therapeutics—9 h-12 h	0.33	0.15
Share of Supplementary Measures—9 h-12 h	0.08	0.03
Share of Diagnostics—15 h-22 h	0.06	0.02
Share of Imaging—15 h-22 h	0.13	0.05
Share of Surgery—15 h-22 h	0.18	0.15
Share of Medication—15 h-22 h	0.01	0.00
Share of Non-surgical Therapeutics—15 h-22 h	0.25	0.09
Share of Supplementary Measures—15 h-22 h	0.04	0.01
Time of Diagnostics—Average	11.15	11.09
Time of Imaging—Average	11.94	11.91
Time of Surgery—Average	10.74	10.96
Time of Medication—Average	11.31	11.50
Time of Non-surgical Therapeutics—Average	11.63	11.28
Time of Supplementary Measures—Average	11.18	10.86
Time of Diagnostics—Variance	12.19	11.79
Time of Imaging—Variance	13.93	14.96
Time of Surgery—Variance	10.36	11.79
Time of Medication—Variance	23.37	21.98
Time of Non-surgical Therapeutics—Variance	14.65	15.67
Time of Supplementary Measures—Variance	17.55	17.92
Urgency		
Share of Patients—Emergency Admission	0.34	0.28
Urgency Score—Variance	0.05	0.05
Urgency Score—Median	0.37	0.22
Urgency Score—75th Percentile	0.49	0.35
Urgency Score—25th Percentile	0.26	0.14
Case timestamps		
Share of Admissions—23 h-4 h	0.03	0.02
Share of Admissions—Weekend	0.13	0.09
Share of Admissions—6 h-12 h	0.64	0.73
Share of Discharge—23 h-4 h	0.01	0.00
Share of Discharge—Weekend	0.16	0.21
Share of Discharge—12 h-21 h	0.46	0.45
Time of Admission—Variance	16.89	13.71
Time of Admission—Average	10.52	9.52
Time of Discharge—Variance	7.44	6.32
Length of Stay—Average	16.34	6.78
Length of Stay—Variance	289.70	122.62
Procedure timestamps		
Duration of Procedure to Discharge—25th Percentile	161.95	44.14
Share of Procedure—23 h-4 h	0.04	0.04
Share of Procedure—Weekend	0.06	0.05
Share of Procedure—6 h-12 h	0.58	0.55
Share of Procedure—12 h-21 h	0.36	0.40
Duration of Admission to Procedure—Average	105.60	53.93
Duration of Procedure to Discharge—Average	317.53	123.71
Duration of Procedure to Discharge—Variance	133873.05	44063.76
Departments		
Days of pre-stationary care—Average	0.27	0.35
Days of post-stationary care—Average	0.23	0.27
Share of Transfers—11 h-4 h	0.03	0.01
Share of Transfers—Weekend	0.14	0.05
Share of Transfers—6 h-12 h	0.20	0.07
Share of Transfers—Different med. Fields	0.24	0.09
No. of Distinct Depts.—Average	1.32	1.11
Share of Time—Intensive Care	0.05	0.02
Share of Transfers—Intensive Care	0.13	0.03
Share of Time—Transfers Different Fields	0.18	0.13
Share of Time—Transfers	0.37	0.23
Diagnoses		
No. of Distinct Main Diagnosis—Total	128.73	183.01
No. of Distinct Secondary Diagnosis—Total	991.04	1013.40
Main Diagnoses—Variation	0.20	0.17
Secondary Diagnoses—Variation	0.01	0.02
Share of Patients—Increased Med. Care	0.33	0.20
Share of Patients—Inpatient ICD	0.32	0.15
Ventilation		
Hours of mechanical ventilation—Average	45.26	5.10
Hours of mechanical ventilation—Variance	23,566.30	5018.85
Hours of mechanical ventilation—25th Percentile	16.87	0.00
Frequency		
Number of Cases	1909	5011

**Table 5:** Definition of feature variables

Definition of feature variables	
Variable	Definition
Complications	
Based on additional data from statutory health insurance companies	
Share of Patients—Painkiller (30-day)	*Percentage of patients with prescription of ATC-Code M01,N02a,N02b in 30-days after discharge*
Share of Patients—Outpatient Emergency (30-day)	*Percentage of patients with visit to outpatient emergency department or visit to physician while out of office hours in 30-days after discharge*
Share of Patients—Readmission (30-day)	*Percentage of patients with all-cause readmission to any hospital in 30-days after discharge*
Share of Patients—In-hospital Mortality	*Percentage of patients discharged as dead*
Comorbidities	
Elixhauser comorbidities and score based on Gasparini A (2018). “comorbidity: An R package for computing comorbidity scores.” *Journal of Open Source Software*, 3, 648. https://doi.org/10.21105/joss.00648	
Elixhauser Score—Average	*Average Elixhauser score (Quan *et al*. 2005) of all cases/patients*
Share of Patients—CHF	*Percentage of patients with congestive heart failure*
Share of Patients—Carit	*Percentage of patients with cardiac arrhythmias*
Share of Patients—Valv	*Percentage of patients with valvular disease*
Share of Patients—PCD	*Percentage of patients with pulmonary circulation disorders*
Share of Patients—PVD	*Percentage of patients with peripheral vascular disorders*
Share of Patients—Hypunc	*Percentage of patients with hypertension, uncomplicated*
Share of Patients—Hypc	*Percentage of patients with hypertension, complicated*
Share of Patients—Para	*Percentage of patients with paralysis*
Share of Patients—Ond	*Percentage of patients with other neurological disorders*
Share of Patients—CPD	*Percentage of patients with chronic pulmonary disease*
Share of Patients—Diabunc	*Percentage of patients with diabetes, uncomplicated*
Share of Patients—Diabc	*Percentage of patients with diabetes, complicated;*
Share of Patients—Hypothy	*Percentage of patients with hypothyroidism*
Share of Patients—RF	*Percentage of patients with renal failure*
Share of Patients—LD	*Percentage of patients with liver disease*
Share of Patients—PUD	*Percentage of patients with peptic ulcer disease, excluding bleeding;*
Share of Patients—AIDS	*Percentage of patients with AIDS/HIV*
Share of Patients—Lymph	*Percentage of patients with lymphoma*
Share of Patients—Metacanc	*Percentage of patients with metastatic cancer*
Share of Patients—Solidtum	*Percentage of patients with solid tumor, without metastasis*
Share of Patients—Rheumd	*Percentage of patients with rheumatoid arthritis/collaged vascular disease*
Share of Patients—Coag	*Percentage of patients with coagulopathy*
Share of Patients—Obes	*Percentage of patients with obesity*
Share of Patients—Wloss	*Percentage of patients with weight loss*
Share of Patients—Fed	*Percentage of patients with fluid and electrolyte disorders*
Share of Patients—Blane	*Percentage of patients with blood loss anemia*
Share of Patients—Dane	*Percentage of patients with deficiency anemia*
Share of Patients—Alcohol	*Percentage of patients with alcohol abuse*
Share of Patients—Drug	*Percentage of patients with drug abuse*
Share of Patients—Psycho	*Percentage of patients with psychoses*
Share of Patients—Depre	*Percentage of patients with depression*
Age—Average	*Average age of all patients*
Share of Patients—Functional Disorders	*Percentage of patients with one of the following ICD coded in the hospital case* *U50.40 Severe motor function impairment* *:* *Barthel Index: 20–35 points* *U50.41 Severe motor function impairment: Motor FIM: 31–42 points* *U50.50 Very severe motor function impairment* *:* *Barthel Index: 0–15 points* *U50.51 Very severe motor function impairment: Motor FIM: 13–30 points* *U51.20 Severe cognitive impairment: Extended Barthel Index: 0–15 points* *U51.21 Severe cognitive impairment: Cognitive FIM: 5–10 points* *U51.22 Severe cognitive impairment: MMSE: 0–16 points*
Assistance	
Share of Discharges to Hospice, Rehab, Nursing	*Percentage of patients with discharge to hospice, rehabilitation facility or nursing care facility*
Share of Patients—Nursing Assistance Level 1	*Percentage of patients with specific degree of long-term care needed (level 1—minor impairment of independence)*
Share of Patients—Nursing Assistance Level 2	*Percentage of patients with specific degree of long-term care needed (level 2—significant impairment of independence)*
Share of Patients—Nursing Assistance Level 3	*Percentage of patients with specific degree of long-term care needed (level 3—Severe impairment of independence)*
Share of Patients—Nursing Assistance Level 4	*Percentage of patients with specific degree of long-term care needed (level 4—Most severe impairment of independence)*
Share of Patients—Nursing Assistance Level 5	*Percentage of patients with specific degree of long-term care needed (level 5—Most severe impairment of independence with special requirements for nursing care)*
Additional Procedures	
Number of Procedures—Average	*Average number of distinct procedures of all cases/patients (includes selected procedure code itself)*
Share of Diagnostics—23 h-5 h	*Percentage of patients with diagnostic procedures between 11 p.m. and 5 a.m*
Share of Imaging—23 h-5 h	*Percentage of patients with imaging procedures between 11 p.m. and 5 a.m*
Share of Surgery—23 h-5 h	*Percentage of patients with surgical procedures between 11 p.m. and 5 a.m*
Share of Medication—23 h-5 h	*Percentage of patients with procedure code for administration of medication between 11 p.m. and 5 a.m*
Share of Non-surgical Therapeutics—23 h-5 h	*Share of patients with non-surgical therapeutic measures between 11 p.m. and 5 a.m*
Share of Supplementary Measures—23 h-5 h	*Percentage of patients with procedure code for supplementary measures between 11 p.m. and 5 a.m*
Share of Diagnostics—9 h-12 h	*Percentage of patients with diagnostic procedures between 9 a.m. and noon*
Share of Imaging—9 h-12 h	*Percentage of patients with imaging procedures between 9 a.m. and noon*
Share of Surgery—9 h-12 h	*Percentage of patients with surgical procedures between 9 a.m. and noon*
Share of Medication—9 h-12 h	*Percentage of patients with procedure code for administration of medication between 9 a.m. and noon*
Share of Non-surgical Therapeutics—9 h-12 h	*Percentage of patients with non-surgical therapeutic measures between 9 a.m. and noon*
Share of Supplementary Measures—9 h-12 h	*Percentage of patients with procedure code for supplementary measures between 9 a.m. and noon*
Share of Diagnostics—15 h-22 h	*Percentage of patients with diagnostic procedures between 3 p.m. and 10 p.m*
Share of Imaging—15 h-22 h	*Percentage of patients with imaging procedures between 3 p.m. and 10 p.m*
Share of Surgery—15 h-22 h	*Percentage of patients with surgical procedures between 3 p.m. and 10 p.m*
Share of Medication—15 h-22 h	*Percentage of patients with procedure code for administration of medication between 3 p.m. and 10 p.m*
Share of Non-surgical Therapeutics—15 h-22 h	*Percentage of patients with non-surgical therapeutic measures between 3 p.m. and 10 p.m*
Share of Supplementary Measures—15 h-22 h	*Percentage of patients with procedure code for supplementary measures between 3 p.m. and 10 p.m*
Time of Diagnostics—Average	*Average time of diagnostic procedure (hour)*
Time of Imaging—Average	*Average time of imaging procedure (hour)*
Time of Surgery—Average	*Average time of surgical procedure (hour)*
Time of Medication—Average	*Average time of procedure code for administration of medication (hour)*
Time of Non-surgical Therapeutics—Average	*Average time of non-surgical therapeutics measure (hour)*
Time of Supplementary Measures—Average	*Average time of procedure code for supplementary measures (hour)*
Time of Diagnostics—Variance	*Variance of time of diagnostic procedure hour*
Time of Imaging—Variance	*Variance of time of imaging procedure (hour)*
Time of Surgery—Variance	*Variance of time of surgical procedure (hour)*
Time of Medication—Variance	*Variance of time of procedure code for administration of medication (hour)*
Time of Non-surgical Therapeutics—Variance	*Variance of time of non-surgical therapeutics measure (hour)*
Time of Supplementary Measures—Variance	*Average time of procedure code for supplementary measures (hour)*
Urgency	
Urgency scores calculated based on Krämer, J., Schreyögg, J. & Busse, R. Classification of hospital admissions into emergency and elective care: a machine learning approach. Health Care Manag Sci 22, 85–105 (2019). https://doi.org/10.1007/s10729-017-9423-5	
Share of Patients—Emergency Admission	*Percentage of patients admitted as emergency/ without referral (not necessarily with high medical urgency)*
Urgency Score—Variance	*Variance of urgency score*
Urgency Score—Median	*Median of urgency score*
Urgency Score—75th Percentile	*75th percentile of urgency score*
Urgency Score—25th Percentile	*25th percentile of urgency score*
Case timestamps	
Share of Admissions—23 h-4 h	*Percentage of patients with admission between 11 p.m. and 4 a.m*
Share of Admissions—Weekend	*Percentage of patients with admission on weekends*
Share of Admissions—6 h-12 h	*Percentage of patients with admission between 11 p.m. and 4 a.m*
Share of Discharge—23 h-4 h	*Percentage of patients with discharge between 11 p.m. and 4 a.m*
Share of Discharge—Weekend	*Percentage of patients with discharge on weekends*
Share of Discharge—12 h-21 h	*Percentage of patients with discharge between 11 p.m. and 4 a.m*
Time of Admission—Variance	*Variance of time of admission (hour)*
Time of Admission—Average	*Average of time of admission (hour)*
Time of Discharge—Variance	*Variance of time of discharge (hour)*
Length of Stay—Average	*Average length of stay (days)*
Length of Stay—Variance	*Variance of length of stay (days)*
Procedure timestamps	
Duration of Procedure to Discharge—25th Percentile	*25th percentile Time from the provision of the procedure to discharge of patients (hours)*
Share of Procedure—23 h-4 h	*Share of procedures with provision between 11 p.m. and 4 a.m*
Share of Procedure—Weekend	*Share of procedures with provision on weekends*
Share of Procedure—6 h-12 h	*Share of procedures with provision between 6 a.m. and noon*
Share of Procedure—12 h-21 h	*Share of procedures with provision between noon and 9 p.m*
Duration of Admission to Procedure—Average	*Average time from admission to the provision of the procedure (hours)*
Duration of Procedure to Discharge—Average	*Average time from the provision of the procedure to discharge of patients (hours)*
Duration of Procedure to Discharge—Variance	*Variance of time from the provision of the procedure to discharge of patients (hours)*
Departments	
Days of pre-stationary care—Average	*Average number of days with pre-stationary care*
Days of post-stationary care—Average	*Average number of days with post-stationary care*
Share of Transfers—11 h-4 h	*Percentage of patients with transfers to other departments (other than the department of admission) between 11 p.m. and 4 a.m*
Share of Transfers—Weekend	*Percentage of patients with transfers to other departments (other than the department of admission) on weekends*
Share of Transfers—6 h-12 h	*Percentage of patients with transfers to other departments (other than the department of admission) between 6 a.m. and noon*
Share of Transfers—Different med. Fields	*Percentage of patients with transfers to departments of other medical specialty (e.g. surgery department and urology)*
No. of Distinct Depts.—Average	*Average number of distinct departments in a case*
Share of Time—Intensive Care	*Average time spent in intensive care department as percentage of all time between admission and discharge*
Share of Transfers—Intensive Care	*Percentage of patients with transfers to intensive care department*
Share of Time—Transfers Different Fields	*Average time spent in department with different specialty than department of admission as percentage of all time between admission and discharge*
Share of Time—Transfers	*Average time spent in department other than department of admission as percentage of all time between admission and discharge*
Diagnoses	
No. of Distinct Main Diagnosis—Total	*Total number of distinct main diagnoses for a procedure code*
No. of Distinct Secondary Diagnosis—Total	*Total number of distinct secondary diagnoses for a procedure code*
Main Diagnoses—Variation	*Variation of main diagnoses for a procedure code, calculated as Herfindahl–Hirschman index*
Secondary Diagnoses—Variation	*Variation of secondary diagnoses for a procedure code as Herfindahl–Hirschman index*
Share of Patients—Increased Med. Care	*Percentage of patients with a diagnosis which requires an increased perioperative effort and possibly also an increased need for follow-up care.. Definition according to expert report * *§ 115b Abs. 1a SGB V*
Share of Patients—Inpatient ICD	*Percentage of patients with a diagnosis which, due to their acute nature, justify an inpatient stay even if a procedure that could in principle be carried out on an outpatient basis was performed. Definition according to expert report * *§ 115b Abs. 1a SGB V*
Ventilation	
Hours of mechanical ventilation – Average	*Average duration of mechanical ventilation (hours)*
Hours of mechanical ventilation – Variance	*Variance of duration of mechanical ventilation (hours)*
Hours of mechanical ventilation—25th Percentile	*25th percentile of duration of mechanical ventilation (hours)*

**Table 6:** List of procedure codes by group AOP-catalogue versions

OPS-Group	No. of codes in version 2019	No. of codes in version 2023	No. of codes in version 2024
Neurological examinations	6	13	13
Biopsy without incision	16	19	80
Biopsy with incision	18	19	27
Diagnostic endoscopy	18	18	21
Exploratory diagnostic measures	1	1	1
Ultrasound examinations	0	1	4
Ultrasound examinations	0	0	1
Imaging of the vascular system	11	11	11
Surgery on the nervous system	63	70	78
Eye surgery	188	238	249
Ear surgery	16	21	21
Nose and sinus surgery	22	22	22
Mouth and facial surgery	60	64	64
Pharynx, larynx, and trachea surgery	10	10	10
Lung and bronchial surgery	1	1	1
Heart surgery	20	51	51
Blood vessel surgery	30	30	37
Hematopoietic and lymphatic system surgery	17	17	17
Digestive tract surgery	84	97	111
Surgery on the urinary organs	24	26	27
Surgery on the male genital organs	33	38	49
Surgery on the female genital organs	55	73	76
Obstetric surgery	4	4	4
Surgery on the jaw and facial bones	13	13	13
Surgery on the musculoskeletal system	1768	1822	1847
Surgery on the breast	11	12	13
Surgery on the skin and subcutaneous tissue	319	322	352
Removal of foreign material and concretions	6	6	6
Manipulations on the digestive tract and urinary tract	5	11	12
Closed reduction of a fracture and joint dislocation	40	40	40
Electrostimulation and electrotherapy	0	0	0
Measures for the blood circulation	20	19	22

**Table 7:** List of procedure codes by group from expert report

Procedure Group	No. of procedures
Neurological examinations	95
Biopsy without incision	222
Biopsy with incision	71
Diagnostic endoscopy	66
Functional tests	25
Exploratory diagnostic measures	31
Other diagnostic measures	36
Ultrasound examinations	17
Ultrasound examination	1
Ultrasound examinations	8
Projection radiography	22
Computed tomography (CT)	7
Optical procedures	10
Imaging of the vascular system	13
Nuclear medicine diagnostic procedures	68
Magnetic resonance imaging (MRI)	15
Other imaging procedures	3
Surgery on the nervous system	88
Surgery on endocrine glands	7
Surgery on the eyes	267
Surgery on the ears	25
Surgery on the nose and paranasal sinuses	28
Surgery on the oral cavity and face	104
Surgery on the pharynx, larynx, and trachea	9
Surgery on the lungs and bronchi	6
Surgery on the heart	120
Surgery on the blood vessels	32
Surgery on the hematopoietic and lymphatic systems	4
Surgery on the digestive tract	107
Surgery on the urinary organs	34
Surgery on the male genital organs	47
Surgery on the female genital organs	88
Obstetric surgery	1
Surgery on the jaw and facial bones	1
Surgery on the musculoskeletal system	207
Surgery on the breast	18
Surgery on the skin and subcutaneous tissue	289
Administration of medication and nutrition and therapeutic injections	10
Removal of foreign material and calculi	33
Manipulation of the digestive tract and urinary tract	17
Other forms of therapeutic catheterization and cannula insertion	4
Dressings	14
Closed reduction of fractures and joint luxation	2
Control of bleeding by tamponade	3
Radiotherapy, nuclear medicine therapy, and chemotherapy	96
Early rehabilitation and physical therapy	7
Electrostimulation and electrotherapy	8
Measures for the respiratory system	4
Measures for the circulatory system	53
Anesthesia and pain therapy	21
Complex treatment	4
Measures during childbirth	8

**Table 8:** Hyperparameters of final model

Parameter	Range	Tuned Value
Number of trees:	1 to 2000	1000
Mtry:	1 to 118	36
Target node size:	2 to 40	40

## Footnotes

1 Both the AOP catalog and the inpatient-only list include a large number of procedures; however, according to the billing data, some are never performed.

## Code availability

The trained model is available in a public repository, as indicated in the body of this manuscript.
