# Supplementary material for: Data-driven identification of outpatient-suitable procedures: a machine learning approach
Source: Health Care Manag Sci. 2026 Mar 23;29(2):14. doi: 10.1007/s10729-026-09758-6 (PMC13009083; doi:10.1007/s10729-026-09758-6)
Supplement: Supplementary file 3 — Supplementary file3 (PDF 2287 KB) [file 10729_2026_9758_MOESM3_ESM.pdf]

SUPPLEMENTARY INFORMATION 1

JOURNAL – HEALTHCARE MANAGEMENT SCIENCE

DATA DRIVEN IDENTIFICATION OF OUTPATIENT-SUITABLE PROCEDURES:

A MACHINE LEARNING APPROACH

SHAP dependence plots

All plots show the 95-%-quintile of data

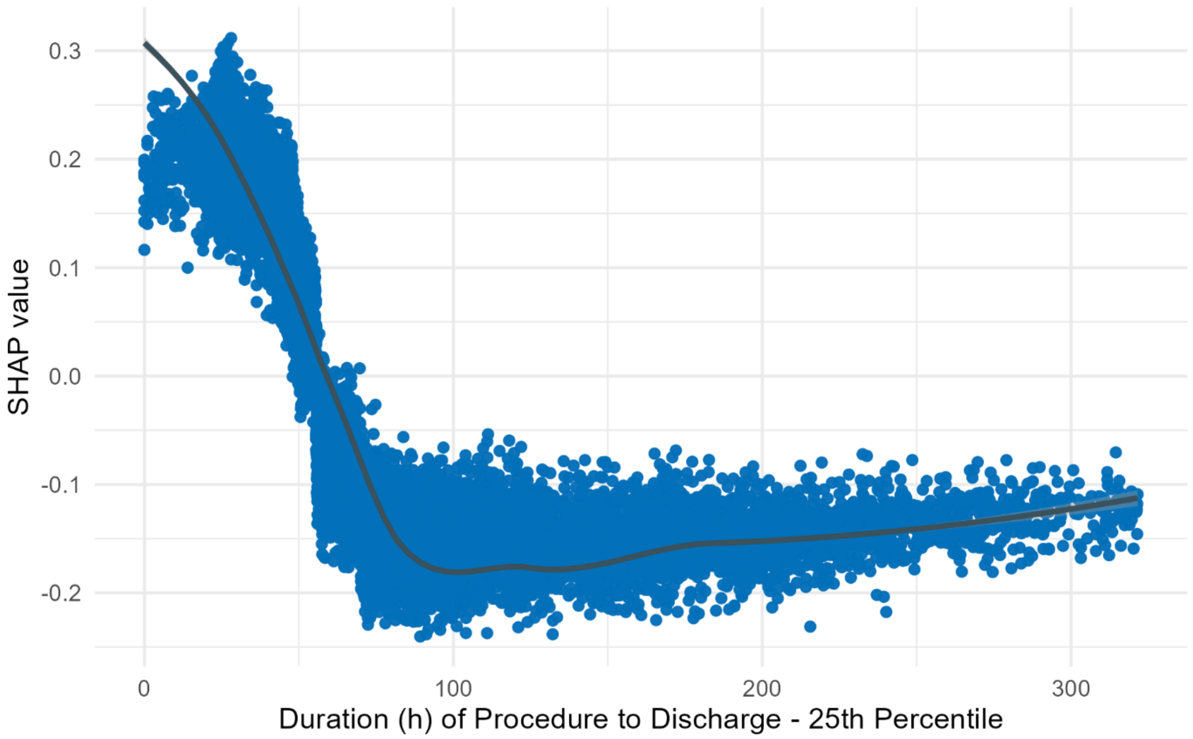

Figure A 1 SHAP dependence plot Duration (h) of Procedure to Discharge - 25th Percentile

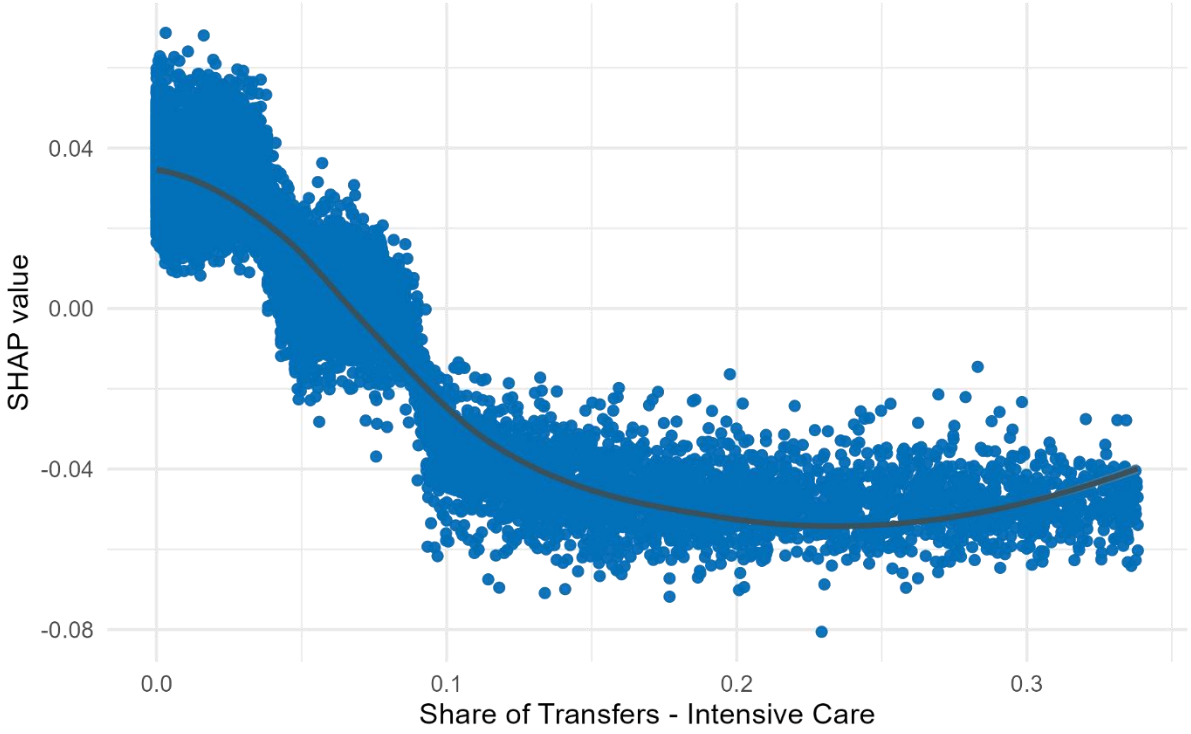

Figure A 2 SHAP dependence plot Share of Transfer – Intensive Care

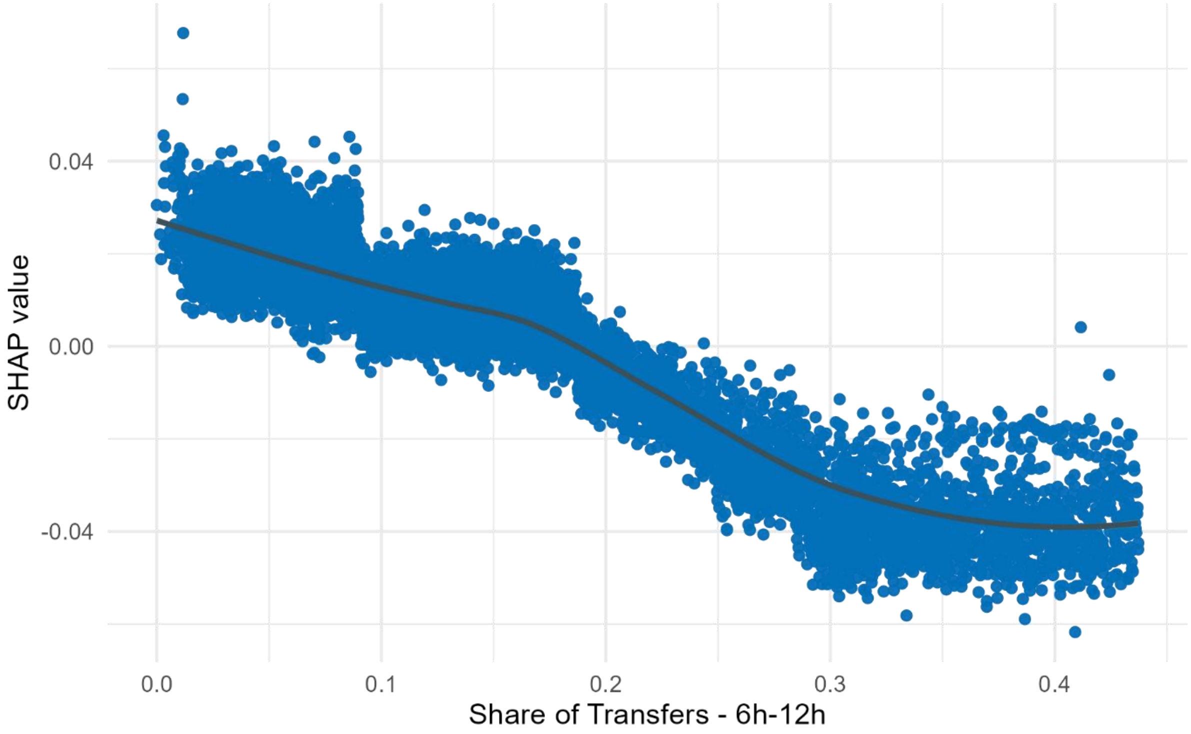

Figure A 3 SHAP dependence plot Share of Transfers - 6h-12h

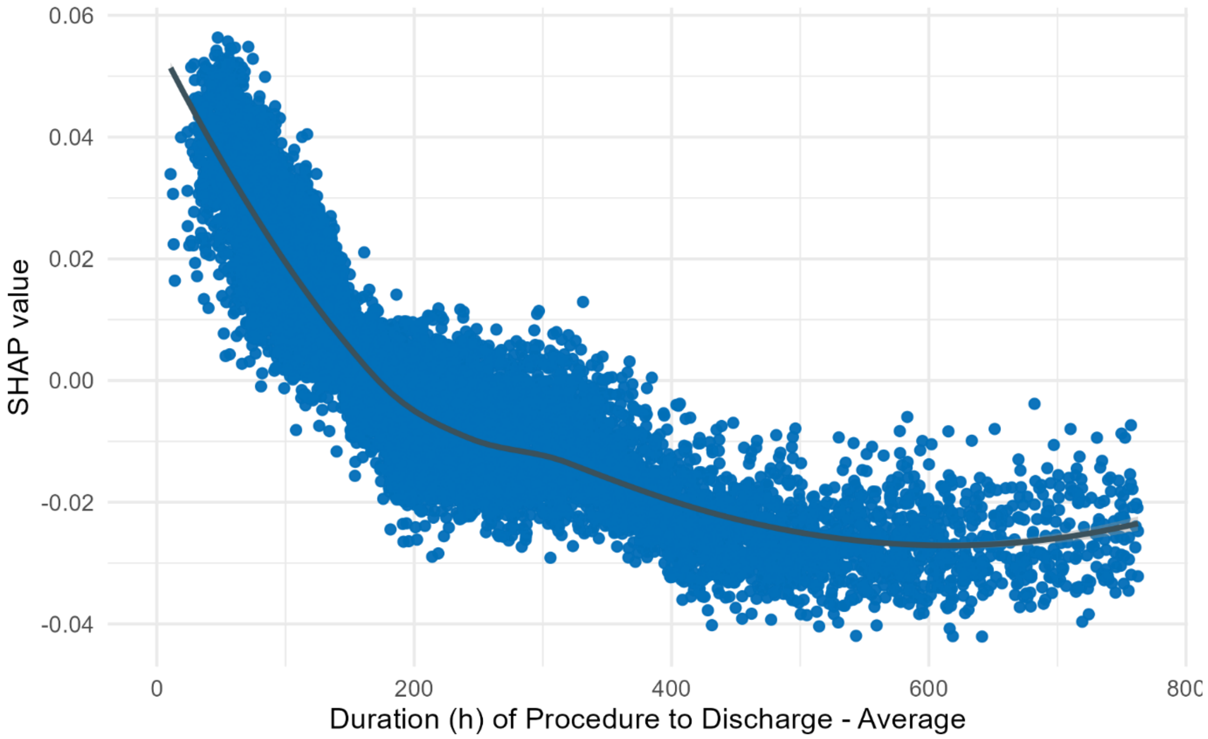

Figure A 4 SHAP dependence plot Duration (h)of Procedure to Discharge – Average

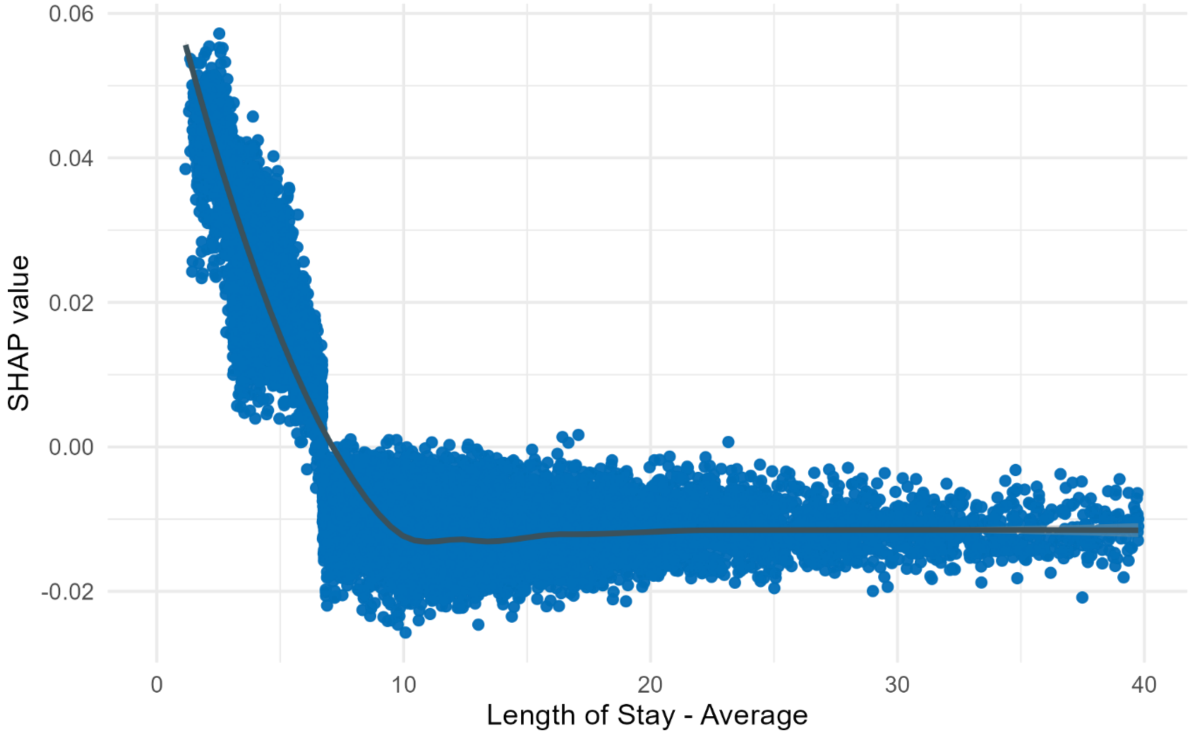

Figure A 5 SHAP dependence plot Length of Stay – Average

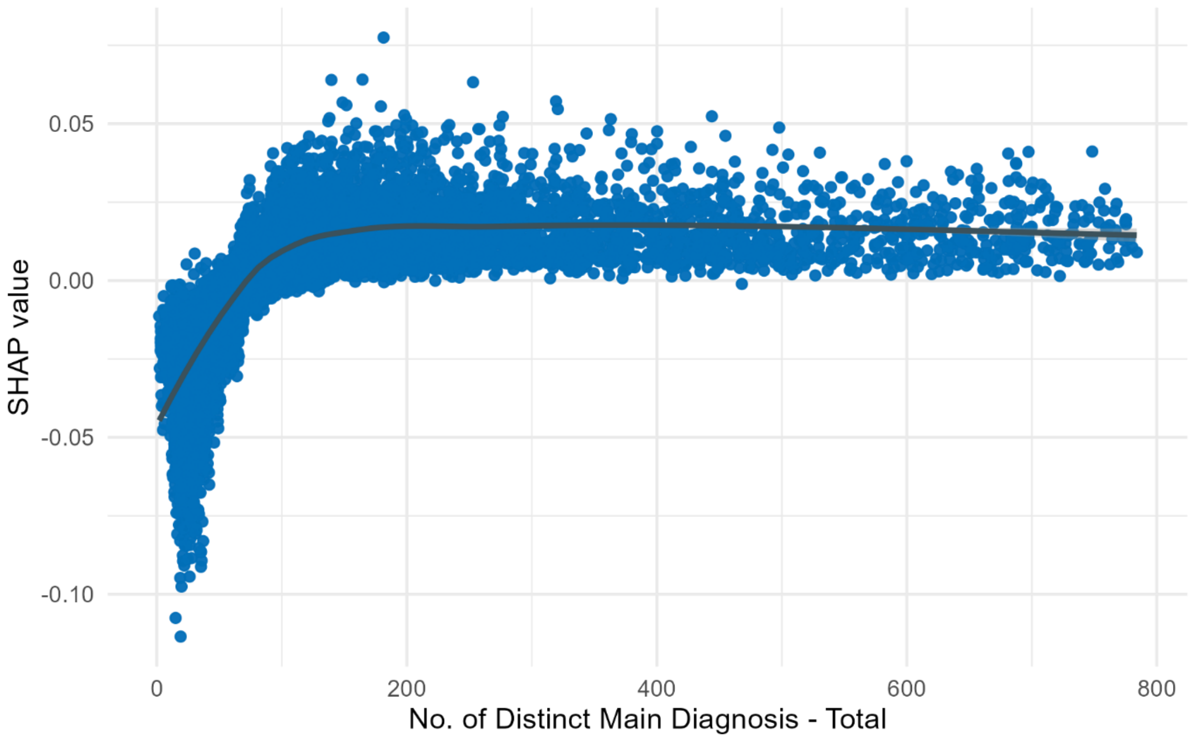

Figure A 6 SHAP dependence plot No. of Distinct Main Diagnosis – Total

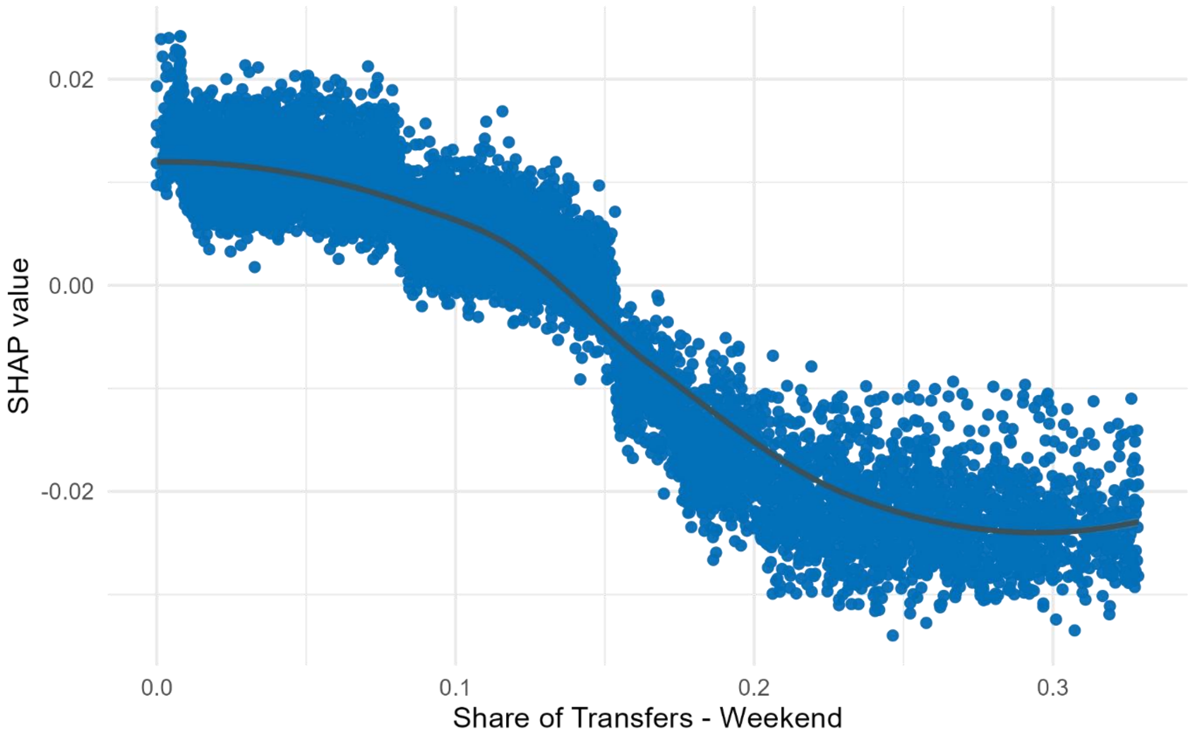

Figure A 7 SHAP dependence plot Share of Transfers - Weekend

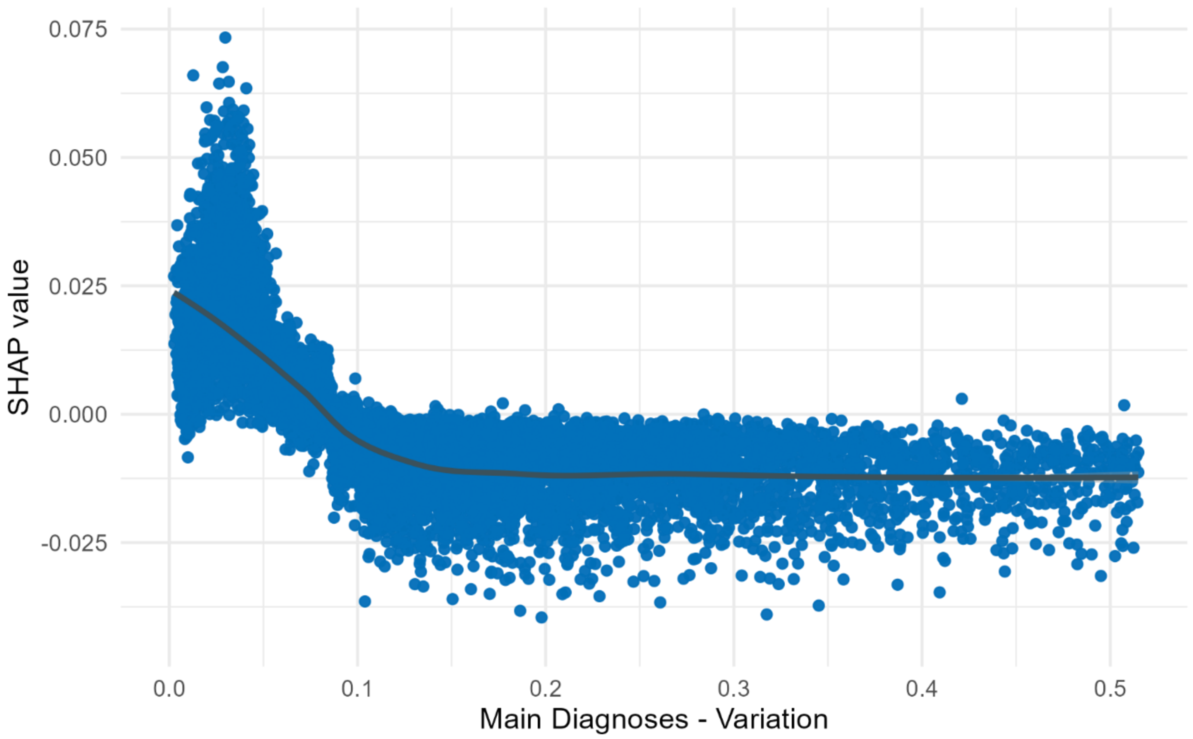

Figure A 8 SHAP dependence plot Main Diagnoses – Variation

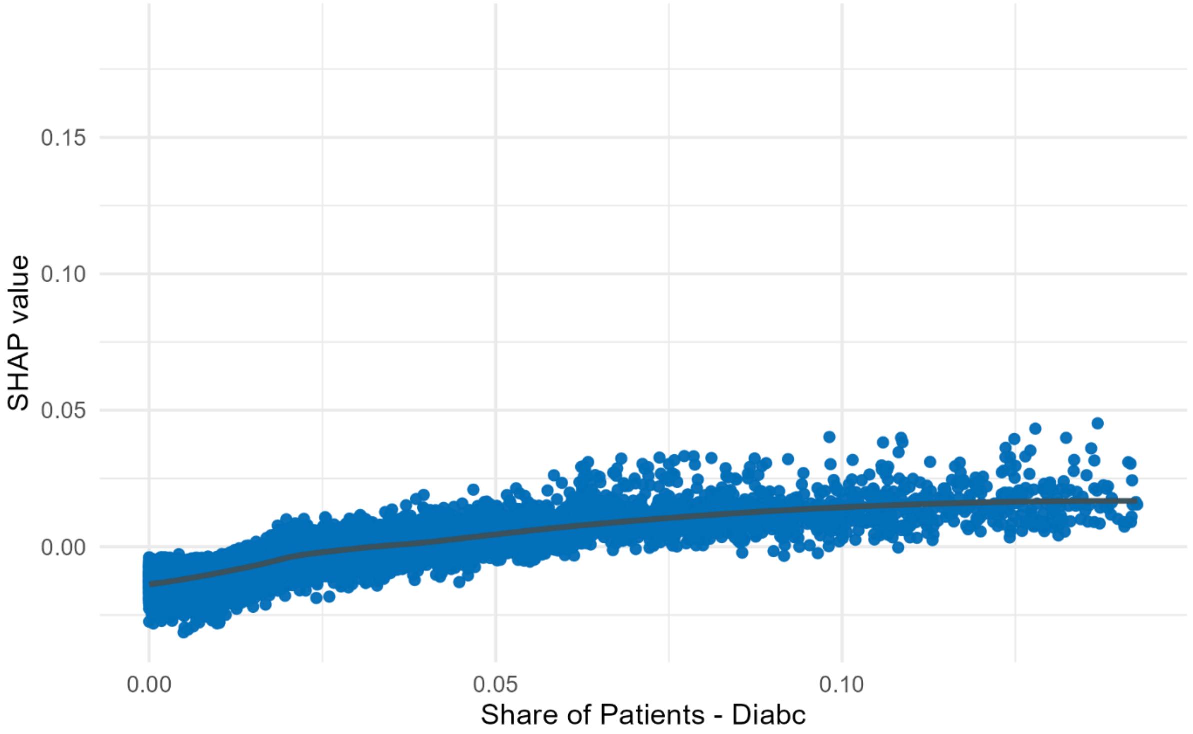

Figure A 9 SHAP dependence plot Share of Patients – Diabc

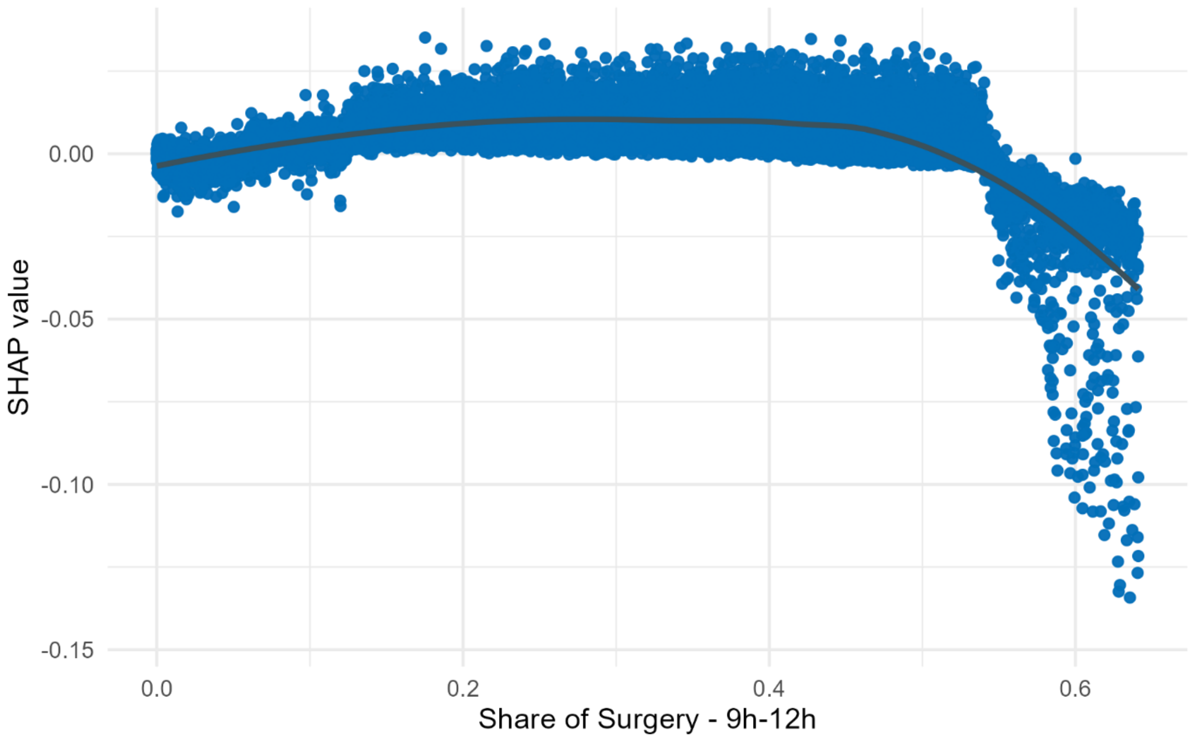

Figure A 10 SHAP dependence plot Share of Surgery from 9-12
